# A Framework for Interpreting Type I Error Rates from a Product‐Term Model of Interaction Applied to Quantitative Traits

**DOI:** 10.1002/gepi.21944

**Published:** 2015-12-14

**Authors:** Tara J. Rao, Michael A. Province

**Affiliations:** ^1^ Division of Statistical GenomicsDepartment of GeneticsWashington University School of MedicineSt. LouisMissouriUnited States of America

**Keywords:** epistasis, qq plots, genomic inflation factor, heteroskedasticity, regression

## Abstract

Adequate control of type I error rates will be necessary in the increasing genome‐wide search for interactive effects on complex traits. After observing unexpected variability in type I error rates from SNP‐by‐genome interaction scans, we sought to characterize this variability and test the ability of heteroskedasticity‐consistent standard errors to correct it. We performed 81 SNP‐by‐genome interaction scans using a product‐term model on quantitative traits in a sample of 1,053 unrelated European Americans from the NHLBI Family Heart Study, and additional scans on five simulated datasets. We found that the interaction‐term genomic inflation factor (lambda) showed inflation and deflation that varied with sample size and allele frequency; that similar lambda variation occurred in the absence of population substructure; and that lambda was strongly related to heteroskedasticity but not to minor non‐normality of phenotypes. Heteroskedasticity‐consistent standard errors narrowed the range of lambda, with HC3 outperforming HC0, but in individual scans tended to create new *P*‐value outliers related to sparse two‐locus genotype classes. We explain the lambda variation as a result of non‐independence of test statistics coupled with stochastic biases in test statistics due to a failure of the test to reach asymptotic properties. We propose that one way to interpret lambda is by comparison to an empirical distribution generated from data simulated under the null hypothesis and without population substructure. We further conclude that the interaction‐term lambda should not be used to adjust test statistics and that heteroskedasticity‐consistent standard errors come with limitations that may outweigh their benefits in this setting.

## Introduction

Genome‐wide studies of interactive effects on complex traits are beginning to appear in the human genetics literature as the potential for interactions to account for trait variation and to shed light on the biological mechanisms underlying complex traits is increasingly appreciated [Figueiredo et al., 2014; Khoury and Wacholder, 2009; Mackay, 2014; Manolio et al., 2009; Thomas, 2010; Thomas et al., 2012; Wei et al., 2014; Wu et al., 2012; Zuk et al., 2012]. Although concern has been raised about the low power to detect such interactions [Manolio et al., 2009; Zuk et al., 2012], maintenance of appropriate type I error rates is a critical first step for any statistical technique so that its results are interpretable. In the genome‐wide association study (GWAS) era, the predominant measures of type I error have been the quantile‐quantile (Q‐Q) plot and the genomic inflation factor λ [Bacanu et al., 2002; The Wellcome Trust Case Control Consortium, 2007]. For a main‐effect scan on a quantitative trait, λ is the asymptotic variance of the test statistics and in practice is typically estimated by comparing the median squared test statistic from a GWAS to the median of the χ^2^ distribution. Lambda usually shows inflation in the presence of population substructure but can be influenced by any artifact that creates bias in test statistics. For genome‐wide tests of interaction, the λ statistic and Q‐Q plot are natural starting points as indicators of type I error rates.

Of many existing methods for detecting interactions in genetic datasets [An et al., 2009; Cordell, 2002, 2009], inference on a product term in the context of a generalized linear model appears to be the most commonly employed. Although product terms can be used to represent four types of epistasis in a model that includes additive and dominance terms [Cheverud, 2000], here we focus on the simpler model $y=\beta_{0}+\beta_{1}x+\beta_{2}z+\beta_{3}xz+ɛ$, where *x* and *z* indicate single‐nucleotide polymorphism (SNP) dosages. While applying this model in a SNP‐by‐genome approach to study gene‐gene interactions in the NHLBI Family Heart Study (FamHS) [Higgins et al., 1996], we found greater variability in type I error rates for the 1‐*df* test on the product term than we expected based on values typically observed in main‐effect GWAS.

A handful of recent works have discussed type I error inflation in gene‐environment interaction scans. Cornelis et al. [2012] observed type I error inflation in a body mass index (BMI)‐by‐genome interaction scan on type 2 diabetes using a product term in a logistic regression. They attribute the inflation to model misspecification. The type I error rate improved when higher orders of BMI were specified, when BMI was dichotomized, or when a sandwich variance estimator was used. Tchetgen and Kraft [2011], referring to the same study, note that misspecification in this model can cause inflation or deflation and recommend Huber's sandwich variance estimator. Voorman et al. [2011] used simulation to show that mean model misspecification or heteroskedasticity can cause Q‐Q plot inflation from an interaction scan on a quantitative trait. They reduced the inflation with a sandwich variance estimator and showed that mean model misspecification causes a similar problem in logistic and Cox regression. Lastly, Almli et al. [2014] jointly tested a SNP and product term in two environment‐by‐genome scans on two quantitative traits related to post‐traumatic stress disorder. Type I error was inflated in all four scans, which they attribute to heteroskedasticity. They reduced it via the Huber‐White sandwich variance estimator and used simulation to show that heteroskedasticity can create Q‐Q plot inflation or deflation.

The sandwich variance estimators were devised to accommodate violations of the homoskedasticity assumption of regression. In a linear regression, heteroskedasticity leaves least‐squares parameter estimates unbiased but renders their standard error estimates inconsistent, which makes inferential tests misleading. The asymptotic heteroskedasticity‐consistent covariance matrix estimator HC0—known synonymously by the names Huber, Eicker, or White, and the first in the family of “sandwich” covariance matrix estimators—is an alternative to the least‐squares covariance matrix estimator and is consistent in the presence of heteroskedasticity [MacKinnon, 2012; White, 1980]. The adjusted estimators HC1–HC3 were developed for use in small samples [Davidson and MacKinnon, 1993; MacKinnon and White, 1985]. The need for adjusted estimators arises because in finite samples HC0 standard errors tend to be biased downward, especially when the regression design contains points of high leverage as occurs in unbalanced designs [Chesher and Jewitt, 1987]. HC3 is adjusted for leverage and has been shown to perform best among HC0‐HC3 [Davidson and MacKinnon, 1993; Long and Ervin, 2000].

Here we place the type I error variability problem into a broader context to better understand its causes and possible solutions including heteroskedasticity‐consistent (HC) standard errors. We focus on gene‐gene interactions though our results have implications for the gene‐environment setting as well. We randomly choose nine SNPs throughout the genome and perform SNP‐by‐genome interaction scans for each of nine quantitative phenotypes on a set of 1,053 unrelated subjects in FamHS. We then perform >400 scans on each of five datasets simulated under the null hypothesis. This allows us to construct empirical distributions of the product‐term λ value and to observe the impact of minor allele frequency (MAF), sample size, stochastically‐occurring heteroskedasticity, phenotype non‐normality and outliers, and HC0 and HC3 standard errors on these distributions.

## Methods

### Family Heart Study

The NHLBI Family Heart Study [Higgins et al., 1996] is a population‐based study begun in 1992 that used participant data from three parent studies to identify and recruit 588 randomly‐sampled families and 657 families with high risk of coronary heart disease. About 6,000 subjects completed a clinic examination between 1994 and 1996 (Visit 1) during which written informed consent was given and a broad range of phenotype information was collected. Fasting blood samples were taken for a variety of laboratory tests as well as for DNA collection. Genotyping of 4,135 European‐American FamHS subjects was performed on Illumina platforms (HumanHap550, Human610‐Quad v1.0, and Human1M‐Duo v3.0) and genotypes were called with BeadStudio (Illumina). Quality control measures included identification of Mendelian errors by LOKI [Heath, 1997] and of incorrect familial relationships using GRR [Abecasis et al., 2001]. Additional SNPs were removed if they were flagged by Illumina, had call rate <98% or MAF < 1%, deviated from Hardy‐Weinberg equilibrium with *P* < 1 × 10^−6^, or were not present in HapMap [The International HapMap Consortium, 2003]. In the present study, we only analyzed autosomal SNPs that met these criteria and were genotyped on all three platforms. This resulted in 493,865 SNPs with MAF ≥ 1% (the cutoff used in Fig. 4A and C) whose MAF distribution is shown in supplementary Figure S1; and 469,763 SNPs with MAF ≥ 5% (the cutoff used for all other FamHS analyses presented here, including main‐effect and two‐locus analyses). The Family Heart Study received IRB approval from each field center; current use of the data is with local IRB approval.

### Statistical Analysis

#### Family Heart Dataset

Only unrelated subjects from Visit 1 of FamHS were analyzed in this study. We selected nine quantitative traits from FamHS for their approximate normality (after log transformation if necessary) and low pairwise correlations. We excluded subjects with diabetes (those with fasting serum glucose ≥ 126 mg/dL or currently taking medications for diabetes). Principal components were calculated on this sample (*n* = 1,130) using EIGENSTRAT [Price et al., 2006]. To choose “repeating” SNPs for SNP‐by‐genome interaction scans, we randomly selected one SNP in each of nine MAF bins (0.05–0.5 in increments of 0.05) from the genotyped, filtered FamHS SNPs. To avoid artifacts in our results due to missing data points, we analyzed only the set of unrelated non‐diabetic subjects who had non‐missing data for all nine phenotypes and all nine repeating SNPs (*n* = 1,053 subjects). Within this dataset, each of the nine phenotypes was adjusted for field center, genotyping platform, and (stepwise with a 5% significance level for staying) age, age^2^, age^3^, and the first 10 principal components. Extreme outliers >4 standard deviations from the mean were adjusted but not used to calculate adjustment parameters. Adjustments were performed separately by sex, and the standardized residuals resulting from the model were used as phenotypes in subsequent analyses. In this final dataset of 1,053 subjects, the nine “repeating” SNPs had low pairwise correlations (all Pearson |*r*| < 0.079) and the nine phenotypes had low pairwise correlations (all Pearson |*r*| < 0.142). Genomic (“non‐repeating”) SNPs in linkage disequilibrium with the nine “repeating” SNPs were not excluded from SNP‐by‐genome interaction analyses.

#### Simulated Datasets

We simulated a total of five datasets and did not include population substructure or SNP effects in any.

**Simulated dataset I: 1,053 subjects, simulated genotypes, FamHS phenotypes**

First, to simulate a dataset with a sample size and allele frequency distribution similar to that of our real FamHS data, we randomly selected 20,000 MAFs from the MAF distribution of FamHS filtered, genotyped SNPs with MAF ≥ 0.05 as calculated in the FamHS dataset of 1,053 subjects. We assumed Hardy‐Weinberg equilibrium to calculate the corresponding genotype frequencies and used the CALL RANTBL routine in SAS to generate genotypes for 1,053 subjects at 20,000 SNPs based on these frequencies. We simulated an additional 46 “repeating” SNPs across a broad frequency spectrum by using a MAF of 0.05–0.5 in increments of 0.01, then following the same procedure as above. To generate phenotypes under the null hypothesis of no SNP effects, we randomly assigned the adjusted FamHS phenotypes to the simulated subjects, keeping the nine phenotype distributions intact but shuffled relative to each other.

**Simulated dataset II: 1,053 subjects, simulated genotypes and phenotypes**
**Simulated dataset III: 5,000 subjects, simulated genotypes and phenotypes**
**Simulated dataset IV: 10,000 subjects, simulated genotypes and phenotypes**

We created three additional datasets (*n* = 1,053, *n* = 5,000, and *n* = 10,000 subjects) by following the same procedure as above for the genotypes, but randomly sampling from a standard normal distribution for each of nine phenotypes using the CALL RANNOR routine in SAS.

**Simulated dataset V: 1,053 subjects, simulated genotypes and 100 phenotypes**

So that we could study a larger number of phenotypes, we created a dataset with 20,000 SNPs and an additional 10 “repeating” SNPs simulated with the same procedure as for the other datasets, but this time using a MAF of 0.05–0.5 in increments of 0.05 to generate the “repeating” SNPs. One hundred phenotypes were simulated by randomly sampling from a standard normal distribution as above.

#### Models

SNPs were coded as 0, 1, or 2 doses of the minor allele in the dataset analyzed. For all SNPs and phenotypes in the one real and five simulated datasets, we fit the following main‐effect‐only model.

(I) $$y=\beta_{0}+\beta_{1}SNP_{\mathrm{non}-\mathrm{repeating}}+ɛ.$$

In the FamHS dataset, we also fit a two‐locus model to calculate λ_2_ values based on β_2_.

(II) $$y=\beta_{0}+\beta_{1}SNP_{\mathrm{repeating}}+\beta_{2}SNP_{\mathrm{non}-\mathrm{repeating}}+ɛ.$$

In all six datasets, we performed SNP‐by‐genome interaction scans by fitting the following product‐term model and conducting a 1‐*df F‐*test of the null hypothesis β_3_ = 0.

(III) $$\begin{array}{ccc} y & = & \beta_{0}+\beta_{1}SNP_{repeating}+\beta_{2}SNP_{\mathrm{non}-\mathrm{repeating}}\\ & & +\;\beta_{3}SNP_{\mathrm{repeating}}\times SNP_{non-repeating}+ɛ. \end{array}$$

We use the term “repeating” for the SNP that is kept the same from pair to pair as the other SNP (“non‐repeating”) proceeds through the genome as in a GWAS. We performed SNP‐by‐genome interaction scans for each of the nine repeating SNPs and nine phenotypes in the FamHS dataset, for a total of 81 scans. In simulated datasets I–IV, we performed scans for each of 46 repeating SNPs and nine phenotypes, for a total of 414 scans per dataset. In simulated dataset V, we performed scans for each of 10 repeating SNPs and 100 phenotypes, totaling 1,000 scans. For each scan we calculated the λ_3_ value as (median *t*_3_^2^)/0.455, where the test statistic *t*_3_ was from the test of β_3_ = 0.

Analyses were implemented with PROC REG in SAS 9.3 (SAS Institute, Cary, NC). Where indicated, HC standard error estimators were used by specifying the HCC and HCCMETHOD = 0 (for HC0) or HCCMETHOD = 3 (for HC3) options in PROC REG. When HC standard errors were used, subjects from two‐locus genotype classes with fewer than five subjects were dropped before the model was applied in order to avoid the worst of the *P*‐value inflation demonstrated in Figure 4; exceptions were Figure 4 in which no minimum cell count was used, and supplementary Figure S13 where, as indicated in the figure, either a minimum of 5 or 25 was used. Applying the minimum cell count did not necessarily mean that all nine two‐locus genotype classes met the minimum cell count, only that each populated genotype class met the minimum cell count.

Finally, to examine type I error rates for the commonly‐used 2‐ and 4‐*df* tests of interaction, we fit model III to simulated datasets II–IV and performed SNP‐by‐genome interaction scans with the 2‐*df* test of β_1_ = β_3_ = 0 as well as the 2‐*df* test of β_2_ = β_3_ = 0 for all 46 repeating SNPs and nine phenotypes. On the same datasets and with the same SNP‐by‐genome approach, we then performed a 4‐*df* test by fitting the model

(IV) $$\begin{array}{ccc} y & = & \beta_{0}+\beta_{1}SNP_{r,a}+\beta_{2}SNP_{r,d}+\beta_{3}SNP_{nr,a}+\beta_{4}SNP_{nr,d}\\ & & +\;\beta_{5}SNP_{r,a}\times SNP_{nr,a}+\beta_{6}SNP_{r,a}\times SNP_{nr,d}\\ & & +\;\beta_{7}SNP_{r,d}\times SNP_{nr,a}+\beta_{8}SNP_{r,d}\times SNP_{nr,d}+ɛ,\; \end{array}$$

where “*a*” indicates an additive (0,1,2) coding, “*d*” indicates a dominance (0,1,0) coding, and “*r*” and “*nr*” indicate the repeating and non‐repeating SNP, respectively. The 4‐*df* test had the null hypothesis of β_5_ = β_6_ = β_7_ = β_8_ = 0; we performed this and the 2‐*df* test as *F* tests using the TEST statement in SAS PROC REG. To allow for a comparison of type I error rates from these tests with those from the 1‐*df* test used in all other analyses, we calculated a λ value for the 2‐ and 4‐*df* tests by finding the χ^2^_1_ values corresponding to the *P*‐values resulting from the *F* test, and dividing the median χ^2^_1_ value by 0.455.

## Results

We chose nine quantitative traits from FamHS for their approximate normality and low pairwise correlations, then adjusted them for covariates (Table 1 and supplementary Fig. S2A). We performed main‐effect‐only scans (model I) on these phenotypes in the FamHS dataset (supplementary Fig. S3) and the resulting λ values ranged from 0.990 to 1.057 (Table 1 and supplementary Table S1). Next we randomly chose one SNP in each of nine MAF bins from the 469,763 genotyped SNPs with MAF ≥ 0.05 (Table 2). Each of these “repeating” SNPs was first fit in a two‐locus model (model II) in which the other SNP progressed through the genome as in a GWAS (“non‐repeating” SNPs) with no interaction term included. The λ_2_ values (corresponding to the non‐repeating SNP) from these 81 two‐locus scans were very similar to those from the main‐effect‐only scans (supplementary Fig. S4).

**Table 1:** Nine FamHS quantitative traits used in SNP‐by‐genome interaction scans and their main‐effect‐only lambda values

Phenotype	Lambda from main‐effect‐only scan
Albumin, serum	1.003
Glucose, serum	0.990
Height	1.057
ln(dietary protein per day)	1.007
ln(fibrinogen, serum)	0.995
ln(LDL cholesterol, serum)	1.015
ln(television hours viewed per day)	1.021
Magnesium, serum	1.006
Potassium, serum	1.010

**Table 2:** Characteristics of repeating SNPs used in SNP‐by‐genome interaction scans

Repeating‐SNP number	Marker name	Chromosome	Number of subjects by genotype: 0/1/2^a^	Minor allele frequency^b^	Gene region/SNP function
1	rs7564315	2	923/126/4	0.06	*KIAA2012*/intron
2	rs10106243	8	840/202/11	0.11	‐
3	rs6065298	20	751/280/22	0.15	‐
4	rs716982	16	630/366/57	0.23	*RBFOX1*/intron
5	rs861528	14	552/430/71	0.27	*ZFYVE21*/ intron
6	rs2408208	5	451/503/99	0.33	*SLC38A9*/intron
7	rs10507467	13	433/488/132	0.36	‐
8	rs11231017	11	376/506/171	0.40	*SCGB1D4*/2.4 kb downstream
9	rs998731	8	295/537/221	0.46	‐

a Number of subjects with indicated dosage of minor allele, totaling 1,053 subjects.

b MAF in FamHS sample of 1,053 unrelated subjects.

**Figure 1:**
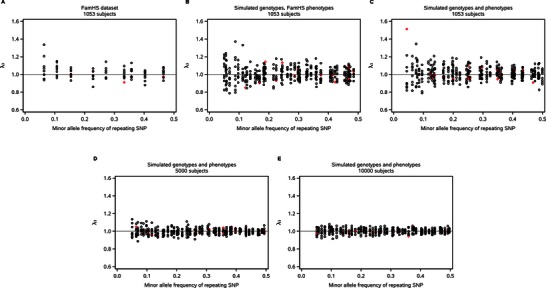
Interaction‐term lambda (λ_3_) from SNP‐by‐genome interaction scans plotted by MAF of repeating SNP. Each data point represents the interaction‐term lambda value from a 1‐*df* SNP‐by‐genome interaction scan for one repeating SNP and one phenotype, for a total of 81 data points (nine repeating SNPs, nine phenotypes) in the FamHS dataset (panel A) and 414 data points (46 repeating SNPs, nine phenotypes) in the simulated datasets (panels B–E, corresponding to simulated datasets I–IV, respectively). Genome size was 469,763 in FamHS and 20,000 in simulated datasets. Simulated datasets were generated without SNP effects or population substructure. Red diamonds indicate that the repeating SNP showed a main effect with *P* < 0.05 in a main‐effect‐only model within the same dataset; black circles indicate all other data points.

Finally, we performed SNP‐by‐genome interaction scans (model III) for each of the nine repeating SNPs and nine phenotypes for a total of 81 scans. The resulting λ_3_ values (corresponding to the product term) ranged from 0.860 to 1.336 (Fig. 1A and supplementary Table S2). The variability in λ_3_ was greatest at low MAF of the repeating SNP. Repeating SNPs that had a main‐effect‐only association at the 0.05 level within the same dataset showed a similar distribution of λ_3_ values as those that did not. We plotted λ_3_ by phenotype (supplementary Fig. S5) and found that all nine phenotypes showed λ_3_ inflation and deflation. To describe the distribution by another measure of type I error, we calculated observed alpha values at the 0.05 level and found a range of 0.035–0.086.

We used simulation to further explore these patterns and possible explanations for them. First, we simulated a dataset of 1,053 subjects and 20,000 SNPs with a similar MAF distribution as FamHS, using the same phenotype values as the real dataset (simulated dataset I; see Methods). We did not include any SNP effects or population substructure. We performed SNP‐by‐genome interaction scans for each of 46 repeating SNPs (one in each of 46 MAF bins from 0.05 to 0.5) for a total of 414 scans on this dataset. Despite the absence of population substructure in the simulated dataset, the resulting λ_3_ values were very consistent with those from the real dataset (Fig. 1B). Next we simulated a similar dataset whose phenotypes were sampled from a standard normal distribution instead of real phenotypes (simulated dataset II). SNP‐by‐genome interaction scans on this dataset yielded a λ_3_ pattern very similar to that from the real dataset and simulated dataset I (Fig. 1C). Because simulated dataset I had more phenotypic outliers as well as non‐normality (as measured by skewness and kurtosis) than simulated dataset II (supplementary Fig. S2A vs. B and supplementary Fig. S6A and B vs. C and D) but gave a similar λ_3_ distribution, outliers and non‐normality did not appear to play a major role in the observed λ_3_ variation. In addition, there was no apparent relationship between λ_3_ and phenotype non‐normality in either dataset (supplementary Fig. S6). In order to examine λ_3_ patterns for a larger number of phenotypes, we analyzed a simulated dataset of sample size 1,053 with 100 phenotypes drawn from a standard normal distribution (simulated dataset V) and found a λ_3_ distribution similar to that for simulated datasets I and II (supplementary Fig. S7). We investigated the influence of sample size on λ_3_ by simulating two larger datasets (5,000 and 10,000 subjects, each with 20,000 SNPs) under the null hypothesis and with nine phenotypes sampled from a standard normal distribution (simulated datasets III and IV). We found that the distribution of λ_3_ narrowed as the sample size increased (Fig. 1D and E). As in the smaller datasets, the λ_3_ distribution narrowed as the MAF of the repeating SNP increased, and repeating SNPs that had a main‐effect‐only association showed λ_3_ values similar to those that did not. For the FamHS dataset as well as all five simulated datasets, λ_3_ values spanned a broader range than the main‐effect‐only λ values on the same dataset (supplementary Tables S1 and S2).

A closer look at the interaction test statistic distributions from individual SNP‐by‐genome scans in FamHS and simulated datasets I–IV showed minor deviations from normality that appeared stochastic (supplementary Fig. S8). The variance of the distributions deviated further from one at smaller sample size. Within the simulated datasets, these empirically‐observed variances were less strongly correlated with λ_3_ as the sample size increased, suggesting that noncentrality in the test statistic distributions became relatively more important to λ_3_ variation at these larger sample sizes (supplementary Fig. S9).

**Figure 2:**
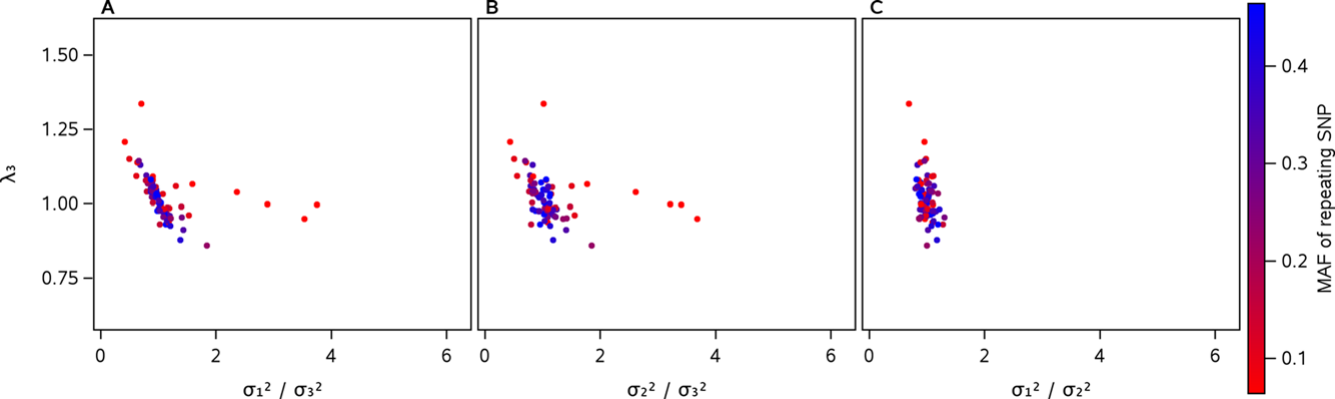
Interaction‐term lambda (λ_3_) from SNP‐by‐genome interaction scans on FamHS dataset plotted by repeating‐SNP genotypic variance ratios. Genotype classes of the repeating SNPs used in SNP‐by‐genome scans in the FamHS dataset are indicated on the *x*‐axes by the number of subjects they contained, where the class with the greatest number of subjects is labeled 1 and the class with the fewest subjects is labeled 3; for example, for repeating SNP 1 (Table 2), the genotype class with 923 subjects is labeled 1, the class with 126 subjects is labeled 2, and the class with four subjects is labeled 3. For each of nine phenotypes, the variance of the phenotype within each of these classes (the “genotypic variance”) was calculated, then all three ratios of the three variances were calculated. The *x*‐axis of panel A indicates the genotypic variance ratio of the largest to the smallest genotype class of the repeating SNP; panel B shows the genotypic variance ratio for the middle to the smallest genotype class of the repeating SNP; and panel C shows the genotypic variance ratio for the largest to the middle genotype class of the repeating SNP. Interaction‐term lambda values from all 81 SNP‐by‐genome interaction scans performed on the FamHS dataset (nine repeating SNPs, nine phenotypes) are indicated by the *y*‐axis.

We next considered the role of heteroskedasticity in λ_3_ variation by examining the three genotypic variance ratios of the repeating SNPs in the FamHS dataset. Even though heteroskedasticity likely occurs for both SNPs in the model, we reasoned that its presence in the repeating SNP would be most influential on λ_3_ because any biases induced by it would be repeated throughout the SNP‐by‐genome scan. Plots of λ_3_ by the variance ratios of the repeating SNP show, first, that the ratios ranged from 0.4 to 3.7, well beyond the assumed ratio of one (Fig. 2). Deviations from one were more common for repeating SNPs with low MAF, and therefore likely resulted from sparseness in the least‐populated genotype classes. When the genotypic variance in the most‐populated genotype class was lower than that in the least‐populated class, λ_3_ was inflated, and when the opposite was true, λ_3_ was deflated. The direction of this relationship between type I error and the variance ratio is the same as that reported in early work on the impact of unequal variances and unequal sample sizes on type I error rates in the *t*‐test [Glass et al., 1972]. Because the same heteroskedasticity occurred for the repeating SNPs in the two‐locus model without an interaction term, yet λ_2_ values from these scans on the non‐repeating SNP were virtually unchanged from the main‐effect λ values (supplementary Fig. S4 vs. Table 1), heteroskedasticity did not appear to significantly affect λ_2_ for the two‐locus model. We hypothesize that it affects λ_3_ for the product‐term model because the product term is a function of the repeating SNP and will partially reflect its variance patterns. We hypothesize that the relationship between λ_3_ and the variance ratios appears strongest for the variance ratio of the two most populated genotype classes because the variance patterns in the product term will best reflect the variance patterns in these classes, while the relationship decays for the least‐populated genotype class of the low‐MAF repeating SNPs because the variance patterns in these sparse cells do not carry over well to the product term once those few subjects are distributed among the three genotypes of the second SNP. To illustrate these patterns at the same sample size but with more data points, we show the same plots for simulated dataset V (supplementary Fig. S10). The relationship between λ_3_ and variance ratios persisted at larger sample sizes (plots for simulated datasets III and IV are shown in supplementary Fig. S11) but here the variance ratios, along with the λ_3_ values, did not range far from one.

**Figure 3:**
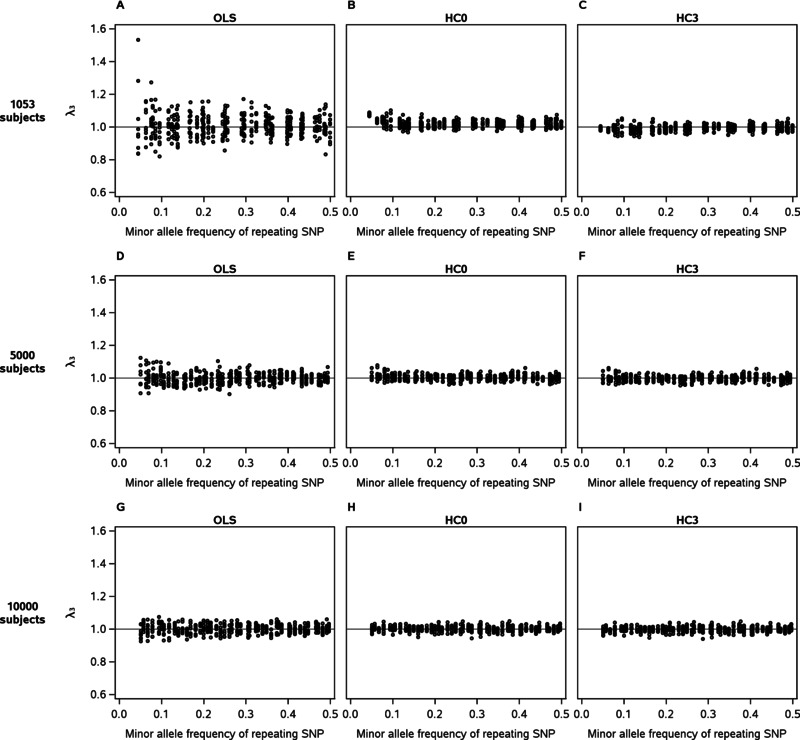
Comparison of interaction‐term lambda (λ_3_) distributions obtained using three types of standard errors in SNP‐by‐genome interaction scans on simulated datasets II–IV. Ordinary least squares standard errors were used in panels A, D, and G; HC0 standard errors were used in panels B, E, and H; and HC3 standard errors were used in panels C, F, and I. Analyses for simulated dataset II are shown in panels A–C; those for simulated dataset III are shown in panels D–F; and those for simulated dataset IV are shown in panels G–I. Each panel contains 414 data points representing SNP‐by‐genome interaction scans for nine phenotypes drawn from a standard normal distribution and 46 repeating SNPs. Subjects in two‐locus genotype classes with fewer than five subjects were dropped before analyses were performed (see Methods and Results), which distinguishes panels A, D, and G from Figure 1 panels C, D, and E, respectively.

Because heteroskedasticity appeared to play an important role in λ_3_ variation, we performed SNP‐by‐genome interaction scans on all datasets using HC0 or HC3 instead of ordinary least squares (OLS) standard errors. In the smallest of simulated datasets II–IV, both HC0 and HC3 standard errors narrowed the range of λ_3_ throughout the repeating‐SNP MAF spectrum but especially at low MAF (Fig. 3A–C). It was also in this MAF range that HC3 standard errors most strongly showed an advantage over HC0 standard errors due to less inflation. By contrast, in the two larger sample sizes, HC standard errors had less impact, and HC3 standard errors showed little improvement over HC0 (Fig. 3D–I). The FamHS dataset and simulated dataset I showed similar results, with HC3 again performing better than HC0 (supplementary Fig. S12).

**Figure 4:**
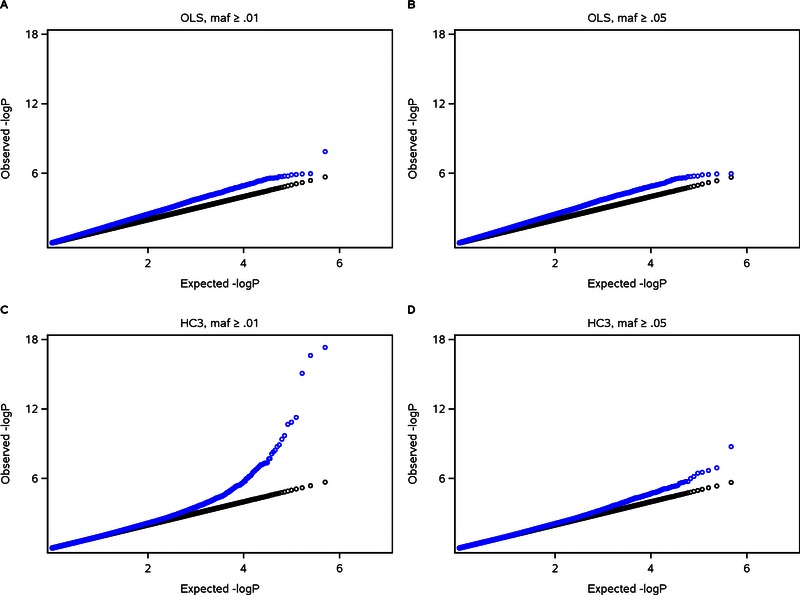
Q‐Q plots from SNP‐by‐genome interaction scans in FamHS dataset for repeating SNP 1 and the phenotype of ln(LDL cholesterol). OLS standard errors are used in panels A and B; HC3 standard errors are used in panels C and D. The non‐repeating SNPs had MAF ≥ 0.01 in panels A and C, and MAF ≥ 0.05 in panels B and D. Repeating SNP 1 had MAF = 0.06 (Table 2). Blue indicates observed vs. expected −log_10_(*P*) values; black indicates the line *y* = *x*.

For the HC0 and HC3 scans, we excluded subjects from two‐locus genotype classes with counts under 5 to avoid the worst of the *P*‐value inflation that we have observed when using HC standard errors on datasets with sparse cells. We reran our original OLS scans using this five‐subject minimum so that fair comparisons could be made with HC0 and HC3, although for OLS this exclusion had little effect on the λ_3_ distributions (e.g., Fig. 1C compared with Fig. 3A). We applied this five‐subject minimum because although HC standard errors narrowed the range of λ_3_ which is based on a median value, in individual Q‐Q plots they sometimes created *P*‐value outliers that did not occur when OLS standard errors were used. This appeared to be related to MAF, so to investigate it further we performed SNP‐by‐genome interaction scans on the FamHS dataset for repeating SNP 1 (MAF = 0.06) using HC3 standard errors, and this time applied a MAF threshold of 0.01 (instead of 0.05) for the non‐repeating SNPs using no minimum cell count. We found a dramatic increase in *P*‐value outliers from these scans when using HC3 but not when using OLS. This occurred for all nine phenotypes but as a representative example we show Q‐Q plots comparing OLS and HC3, using MAF thresholds of 0.01 or 0.05, for one phenotype (Fig. 4). Assuming the *P*‐value outliers were related to sparse cells, we applied a MAF threshold of 0.05 with an additional requirement that a minimum of five subjects occur in each two‐locus genotype class that was populated—so that any subjects in classes more sparse than this were dropped from the dataset before applying the model—and found further improvement in the Q‐Q plot (supplementary Fig. S13B). We examined two‐locus genotype counts corresponding to the *P*‐values at the significant end of the distribution and found that cell counts were more sparse (more likely to have just cleared the cutoff of five subjects) than for *P*‐values at the other end of the distribution. It was only when a minimum of 25 subjects per populated cell was applied that the Q‐Q plot appeared to follow the null distribution (supplementary Fig. S13D). We compared HC0 to HC3 using these minima and found that HC3 showed an improvement over HC0 when a minimum of five subjects was applied, but there was little difference between the two when the minimum was increased to 25 (supplementary Fig. S13).

## Discussion

Our results lead us to the following conclusions.

1. **Interaction‐term test statistics within a SNP‐by‐genome scan are not independent, so the corresponding λ_3_ value is not directly comparable to λ from a main‐effect‐only scan**. The non‐independence arises because the interaction term is the product of one term that repeats and one that does not. As discussed in Kam and Franzese [2007], researchers using the product‐term model of interaction have long been aware that collinearity arises from the inclusion of a product term and its component terms within the same model; this has led to discussions in the social sciences literature about inflation of standard errors and whether centering variables before computing their product alleviates this (which it does not). While this collinearity is not a concern in general, in the setting of a SNP‐by‐genome interaction scan it becomes consequential because the product term is collinear with an additive term that is the same from SNP pair to SNP pair within a scan, making the product terms within a scan correlated with each other. One way to illustrate this is with Q‐Q plots from a two‐locus scan without an interaction term. A Q‐Q plot based on the repeating SNP shows virtually identical results in every regression, but the non‐repeating SNP—which is independent from the repeating SNP in all cases except for a few in linkage disequilibrium with it—has a Q‐Q plot nearly identical to that from the main‐effect scan on the same phenotype (supplementary Fig. S14). When the product term is added to the model, it should show features of both conceptual extremes; the λ_3_ statistic can be expected to have a greater variance than the main‐effect λ even if no bias is present in the interaction test statistics. If there is bias, it will tend to be in the same direction throughout a scan. By contrast, in a main‐effect‐only scan, small biases due to misspecification likely occur in each test but cancel out on average because the tests are virtually independent. We believe that similar biases resulting from random minor violations of regression assumptions (especially heteroskedasticity) that occur as interaction‐term test statistics fail to reach asymptotic properties, coupled with non‐independence within a scan, create the observed λ_3_ variability. These biases may be greater for the interaction model than simpler models because of higher leverage values, as discussed in conclusion 5. We expect this pattern of variation in type I error to occur in any setting in which a test is performed on at least one product term formed using a component term that repeats from test to test. We extended our analysis to the 2‐ and 4‐*df* tests of interaction (models III and IV; supplementary Fig. S15) and the results are consistent with this explanation for variability in type I error, particularly in the stark contrast between type I error rates for a 2‐*df* test on the repeating SNP and product term compared to a 2‐*df* test on the non‐repeating SNP and product term.
  The λ_3_ variability arising in our simulated datasets II–V is the minimal disturbance that can be expected in type I error rates for SNP‐by‐genome interaction scans. While the outliers and minor non‐normality in our real phenotypes did not appear to affect the overall λ_3_ distribution (Fig. 1B vs. C), it is possible in theory for more severe nonlinearity, non‐normality, or other violations of regression assumptions to create further variation in λ_3_. So could population substructure; the expected impact of this on λ_3_ has been briefly explored [Bacanu et al., 2002] but is still not well understood. Finally, linkage disequilibrium can create false positives in tests of interaction [e.g., Hemani et al., 2014a; Wood et al., 2014; Hemani et al., 2014b]. Although these may not be sufficient to affect the overall λ_3_ value, the SNPs proximal to the repeating SNP in a SNP‐by‐genome scan may be best analyzed separately with the possibility of confounding by linkage disequilibrium in mind.
2. **One way to interpret λ_3_ is by comparison to a simulation‐based empirical distribution**. Following the explanation for λ_3_ variability given above, one strategy for interpreting λ_3_ is to compare it to an empirical distribution of λ_3_ generated by simulating a similar‐sized dataset with no SNP effects and without population substructure, and then performing a large number of SNP‐by‐genome scans on it. Here, we would compare our FamHS results in Figure 1A to our simulation results in Figure 1B and conclude that our results occurred within the expected distribution. As the sample size and repeating‐SNP MAF decrease, this approach would begin to become unreasonable; another approach might be to use the expected correlation structure within a scan to calculate an adjusted λ_3_ value, but it is unclear whether this could be done in a way that does not compromise the ability to detect population substructure or other sources of real bias in the data.
3. **The λ_3_ value should not be used to adjust interaction test statistics**. Because some of the apparent bias is due only to non‐independence, “correcting” it with λ_3_ would introduce a new source of bias. Also, heteroskedasticity and other sources of bias likely occur for both SNPs, so for an interaction between two SNPs, two λ_3_ values would become relevant.
4. **If HC standard errors are used, HC3 is preferable to HC0; but both versions come with limitations that may outweigh their benefits in this setting**. In our data, the advantage of HC3 over HC0 was most obvious at low sample size and repeating‐SNP MAF. This is unsurprising because the only difference between HC0 and HC3 is a leverage adjustment, as we discuss in more detail here. For the classical regression model Y=Xβ+ɛ, the OLS solution vector is $\hat{\beta }={\left(X^{\prime }X\right)}^{-1}X^{\prime }y$ with covariance matrix $\mathrm{var}\left(\hat{\beta }\right)={\left(X^{\prime }X\right)}^{-1}X^{\prime }\Omega X{\left(X^{\prime }X\right)}^{-1}$, where $\Omega =E(ɛ{ɛ}^{\prime })$. In OLS, Ω is estimated by pooling the squared residuals $e_{i}^{2}$ over the entire sample, so that ${\hat{\Omega }}_{OLS}=\mathrm{diag}\left[\Sigma_{i=1}^{n}e_{i}^{2}/\left(n-k\right)\right]$ where *k* is the number of parameters in the model. HC0 instead estimates Ω using the squared OLS residual for each individual observation so that ${\hat{\Omega }}_{HC0}=\mathrm{diag}\left[e_{i}^{2}\right]$. HC3 is similar to HC0 but adjusts each observation's squared residual by a function of its leverage value: ${\hat{\Omega }}_{HC3}=\mathrm{diag}\left[e_{i}^{2}/{\left(1-h_{ii}\right)}^{2}\right]$.
  The use of HC standard errors in this setting comes with limitations. One is that HC standard errors do not correct bias in the coefficient estimates themselves, so are an incomplete solution to *P*‐value bias arising from model misspecification when that misspecification is not purely in the form of heteroskedasticity. A recent work from the field of political science [King and Roberts, 2015] points this out and argues that HC standard errors function better as a flag for misspecification than as a default approach taken in anticipation of it.
  Another problem is that although HC standard errors narrowed the range of λ_3_, they came with the trade‐off of creating *P*‐value outliers that appeared to be due to cell sparseness. The equations for HC0 and HC3 suggest an instability to the Ω estimate when a predictor variable is very unbalanced: an entire two‐locus genotype class becomes represented by the residuals from a small handful of subjects. It is also possible that HC3 remains biased downward in some situations despite its leverage adjustment. In either case, the choice of MAF threshold for both predictor variables becomes more important with HC than OLS standard errors, and the appropriate threshold at a given sample size will not necessarily be clear.
  Because a SNP‐by‐genome scan is a hypothesis‐generating tool that targets the significant end of the *P*‐value distribution, this risk that comes with HC errors must be weighed against the benefit. At larger sample size and higher MAF of the repeating SNP, HC standard errors showed little improvement over OLS, so OLS appears to be a better choice; λ_3_ values that range too wide could be compared to a simulation‐based empirical distribution of λ_3_. Unfortunately, the picture is less clear at lower sample size and greater unbalancedness of the predictor variables. These were the conditions under which HC standard errors showed the greatest advantage over OLS in terms of λ_3_ but were also the conditions under which they were most prone to creating *P*‐value outliers. A resampling‐based method might offer an alternative, but would be computationally impractical to perform on this scale.
5. **The product‐term model draws observations to higher points of leverage than a two‐locus model on the same dataset, predicting that inference on the product‐term model is comparatively more sensitive to deviations from the assumptions of the classical regression model**. While examining regression diagnostic plots, we noticed that the product‐term model increased the leverage of some two‐locus genotype classes, especially sparsely‐populated ones, compared to a two‐locus model without an interaction term (supplementary Fig. S16). In a multiple regression, points of high leverage are multivariate outliers of the predictor variables, and have high potential for influence on the coefficient estimates [Belsley et al., 1980]. It is reasonable that the addition of the product term, which by definition is correlated with its component terms, has this effect because few observations can distinguish the predictors from each other. This problem could be similarly expressed as a multicollinearity problem that is worsened by the unbalancedness of predictor variables inherent to genetic datasets. Expressing the problem in terms of leverage has the advantage of making it directly relatable to the regression‐diagnostic setting and to the choice of HC standard errors, some of which are adjusted for leverage.

Ultimately, as genetics consortia begin to search for interactions across multiple datasets, the larger sample sizes obtained will not only improve the power to detect interactions, but also improve the validity of the interaction test via smaller random violations of regression assumptions and smaller leverage values. The apparent validity as viewed through λ_3_ will also improve as sample size increases, because the actual validity improves and because collinearity is reduced as sample size increases, which reduces the non‐independence of interaction test statistics within a scan. For SNP‐by‐genome analyses, these observations favor the use of combined individual‐level data in a “mega” rather than the traditional “meta” analysis approach, which corrects for λ at the study level and sometimes meta‐analysis level [Winkler et al., 2014]. Yet even at larger sample sizes, these studies would benefit from methods to assess and correct for population substructure that are better tailored to the unique setting of genome‐wide interaction testing.

## Supporting information

Disclaimer: Supplementary materials have been peer‐reviewed but not copyedited.

Supporting Information

## References

[gepi21944-bib-0001] Abecasis GR , Cherny SS , Cookson WO , Cardon LR . 2001 GRR: graphical representation of relationship errors. Bioinformatics 17(8):742–743. 11524377 10.1093/bioinformatics/17.8.742

[gepi21944-bib-0002] Almli LM , Duncan R , Feng H , Ghosh D , Binder EB , Bradley B , Ressler KJ , Conneely KN , Epstein MP . 2014 Correcting systematic inflation in genetic association tests that consider interaction effects: application to a genome‐wide association study of posttraumatic stress disorder. JAMA Psychiatry 71(12):1392–1399. 25354142 10.1001/jamapsychiatry.2014.1339 PMC4293022

[gepi21944-bib-0003] An P , Mukherjee O , Chanda P , Yao L , Engelman CD , Huang C‐H , Zheng T , Kovac IP , Dubé M‐P , Liang X and others . 2009 The challenge of detecting epistasis (G×G Interactions): Genetic Analysis Workshop 16. Genet Epidemiol 33(S1):S58–S67. 19924703 10.1002/gepi.20474 PMC3692280

[gepi21944-bib-0004] Bacanu SA , Devlin B , Roeder K . 2002 Association studies for quantitative traits in structured populations. Genet Epidemiol 22(1):78–93. 11754475 10.1002/gepi.1045

[gepi21944-bib-0005] Belsley DA , Kuh E , Welsch RE . 1980 Regression Diagnostics: Identifying Influential Data and Sources of Collinearity. New York: Wiley.

[gepi21944-bib-0006] Chesher A , Jewitt I . 1987 The bias of a heteroskedasticity consistent covariance matrix estimator. Econometrica 55(5):1217–1222.

[gepi21944-bib-0007] Cheverud JM . 2000 *Detecting Epistasis among Quantitative Trait Loci* In: Wolf JB, Brodie ED, Wade MJ, editors. Epistasis and the Evolutionary Process. Oxford; New York: Oxford University Press, pp. 58–81.

[gepi21944-bib-0008] Cordell HJ . 2002 Epistasis: what it means, what it doesn't mean, and statistical methods to detect it in humans. Hum Mol Genet 11(20):2463–2468. 12351582 10.1093/hmg/11.20.2463

[gepi21944-bib-0009] Cordell HJ . 2009 Detecting gene‐gene interactions that underlie human diseases. Nat Rev Genet 10(6):392–404. 19434077 10.1038/nrg2579 PMC2872761

[gepi21944-bib-0010] Cornelis MC , Tchetgen EJT , Liang L , Qi L , Chatterjee N , Hu FB , Kraft P . 2012 Gene‐environment interactions in genome‐wide association studies: a comparative study of tests applied to empirical studies of type 2 diabetes. Am J Epidemiol 175(3):191–202. 22199026 10.1093/aje/kwr368 PMC3261439

[gepi21944-bib-0011] Davidson R , MacKinnon JG . 1993 Estimation and Inference in Econometrics. New York: Oxford University Press.

[gepi21944-bib-0012] Figueiredo JC , Hsu L , Hutter CM , Lin Y , Campbell PT , Baron JA , Berndt SI , Jiao S , Casey G , Fortini B and others . 2014 Genome‐wide diet‐gene interaction analyses for risk of colorectal cancer. PLoS Genet 10(4):e1004228. 24743840 10.1371/journal.pgen.1004228 PMC3990510

[gepi21944-bib-0013] Glass GV , Peckham PD , Sanders JR . 1972 Consequences of failure to meet assumptions underlying the fixed effects analyses of variance and covariance. Rev Educ Res :237–288.

[gepi21944-bib-0014] Heath SC. 1997 Markov chain Monte Carlo segregation and linkage analysis for oligogenic models. Am J Hum Genet 61(3):748–760. 9326339 10.1086/515506 PMC1715966

[gepi21944-bib-0015] Hemani G , Shakhbazov K , Westra HJ , Esko T , Henders AK , McRae AF , Yang J , Gibson G , Martin NG , Metspalu A and others . 2014a Detection and replication of epistasis influencing transcription in humans. Nature 508(7495):249–253. 24572353 10.1038/nature13005 PMC3984375

[gepi21944-bib-0016] Hemani G , Shakhbazov K , Westra HJ , Esko T , Henders AK , McRae AF , Yang J , Gibson G , Martin NG , Metspalu A and others . 2014b Hemani et al. reply. Nature 514(7520):E5–E6. 25279929 10.1038/nature13692 PMC4404158

[gepi21944-bib-0017] Higgins M , Province M , Heiss G , Eckfeldt J , Ellison RC , Folsom AR , Rao DC , Sprafka JM , Williams R . 1996 NHLBI Family Heart Study: objectives and design. Am J Epidemiol 143(12):1219–1228. 8651220 10.1093/oxfordjournals.aje.a008709

[gepi21944-bib-0018] International HapMap C . 2003 The International HapMap Project. Nature 426(6968):789–796. 14685227 10.1038/nature02168

[gepi21944-bib-0019] Kam CD , Franzese RJ . 2007 Modeling and Interpreting Interactive Hypotheses in Regression Analysis. Ann Arbor: University of Michigan.

[gepi21944-bib-0020] Khoury MJ , Wacholder S . 2009 Invited commentary: from genome‐wide association studies to gene‐environment‐wide interaction studies—challenges and opportunities. Am J Epidemiol 169(2):227–230. 19022826 10.1093/aje/kwn351 PMC2727257

[gepi21944-bib-0021] King G , Roberts ME . 2015 How robust standard errors expose methodological problems they do not fix, and what to do about it. Polit Anal 23(2):159–179.

[gepi21944-bib-0022] Long JS , Ervin LH . 2000 Using heteroscedasticity consistent standard errors in the linear regression model. Am Stat 54(3):217–224.

[gepi21944-bib-0023] Mackay TF. 2014 Epistasis and quantitative traits: using model organisms to study gene‐gene interactions. Nat Rev Genet 15(1):22–33. 24296533 10.1038/nrg3627 PMC3918431

[gepi21944-bib-0024] MacKinnon JG . 2012 *Thirty Years of Heteroskedasticity‐Robust Inference* In: Chen X, Swanson NR, editors. Recent Advances and Future Directions in Causality, Prediction, and Specification Analysis: Essays in Honor of Halbert L. White Jr., 1st edition Dordrecht: Springer, pp. 437–461.

[gepi21944-bib-0025] MacKinnon JG , White H . 1985 Some heteroskedasticity‐consistent covariance matrix estimators with improved finite sample properties. J Econometrics 29(3):305–325.

[gepi21944-bib-0026] Manolio TA , Collins FS , Cox NJ , Goldstein DB , Hindorff LA , Hunter DJ , McCarthy MI , Ramos EM , Cardon LR , Chakravarti A and others . 2009 Finding the missing heritability of complex diseases. Nature 461(7265):747–753. 19812666 10.1038/nature08494 PMC2831613

[gepi21944-bib-0027] Price AL , Patterson NJ , Plenge RM , Weinblatt ME , Shadick NA , Reich D . 2006 Principal components analysis corrects for stratification in genome‐wide association studies. Nat Genet 38(8):904–909. 16862161 10.1038/ng1847

[gepi21944-bib-0028] Tchetgen EJT , Kraft P . 2011 On the robustness of tests of genetic associations incorporating gene‐environment interaction when the environmental exposure is misspecified. Epidemiology 22(2):257–261. 21228699 10.1097/EDE.0b013e31820877c5 PMC5972372

[gepi21944-bib-0029] Thomas D. 2010 Gene–environment‐wide association studies: emerging approaches. Nat Rev Genet 11(4):259–272. 20212493 10.1038/nrg2764 PMC2891422

[gepi21944-bib-0030] Thomas DC , Lewinger JP , Murcray CE , Gauderman WJ . 2012 Invited commentary: GE‐Whiz! Ratcheting gene‐environment studies up to the whole genome and the whole exposome. Am J Epidemiol 175(3):203–207. 22199029 10.1093/aje/kwr365 PMC3261438

[gepi21944-bib-0031] Voorman A , Lumley T , McKnight B , Rice K . 2011 Behavior of QQ‐plots and genomic control in studies of gene‐environment interaction. PLoS One 6(5):e19416. 21589913 10.1371/journal.pone.0019416 PMC3093379

[gepi21944-bib-0032] Wei W‐H , Hemani G , Haley CS . 2014 Detecting epistasis in human complex traits. Nat Rev Genet 15(11):722–733. 25200660 10.1038/nrg3747

[gepi21944-bib-0033] Wellcome Trust Case Control C . 2007 Genome‐wide association study of 14,000 cases of seven common diseases and 3,000 shared controls. Nature 447(7145):661–678. 17554300 10.1038/nature05911 PMC2719288

[gepi21944-bib-0034] White H. 1980 A heteroskedasticity‐consistent covariance matrix estimator and a direct test for heteroskedasticity. Econometrica 48(4):817–838.

[gepi21944-bib-0035] Winkler TW , Day FR , Croteau‐Chonka DC , Wood AR , Locke AE , Mägi R , Ferreira T , Fall T , Graff M , Justice AE and others . 2014 Quality control and conduct of genome‐wide association meta‐analyses. Nat Protoc 9(5):1192–1212. 24762786 10.1038/nprot.2014.071 PMC4083217

[gepi21944-bib-0036] Wood AR , Tuke MA , Nalls MA , Hernandez DG , Bandinelli S , Singleton AB , Melzer D , Ferrucci L , Frayling TM , Weedon MN . 2014 Another explanation for apparent epistasis. Nature 514(7520):E3–E5. 25279928 10.1038/nature13691 PMC6478385

[gepi21944-bib-0037] Wu C , Kraft P , Zhai K , Chang J , Wang Z , Li Y , Hu Z , He Z , Jia W , Abnet CC and others . 2012 Genome‐wide association analyses of esophageal squamous cell carcinoma in Chinese identify multiple susceptibility loci and gene‐environment interactions. Nat Genet 44(10):1090–1097. 22960999 10.1038/ng.2411

[gepi21944-bib-0038] Zuk O , Hechter E , Sunyaev SR , Lander ES . 2012 The mystery of missing heritability: genetic interactions create phantom heritability. Proc Natl Acad Sci USA 109(4):1193–1198. 22223662 10.1073/pnas.1119675109 PMC3268279

