# Supplementary material for: A Framework for Interpreting Type I Error Rates from a Product‐Term Model of Interaction Applied to Quantitative Traits
Source: Genet Epidemiol. 2015 Dec 14;40(2):144–53. doi: 10.1002/gepi.21944 (PMC4738444; doi:10.1002/gepi.21944)
Supplement: Supplementary file 1 — Supporting Information [file GEPI-40-144-s001.docx]

**Supporting Information**

**Table S1. Descriptive statistics for λ values from main-effect scans (model I) on all datasets.**

| **Dataset** | **Number of λ values** | **Range of λ values** | **Mean of λ values** | **Standard deviation of λ values** |
| --- | --- | --- | --- | --- |
| FamHS | 9 | 0.990-1.057 | 1.012 | 0.019 |
| Simulated dataset I | 9 | 0.978-1.026 | 1.003 | 0.017 |
| Simulated dataset II | 9 | 0.981-1.018 | 0.997 | 0.012 |
| Simulated dataset III | 9 | 0.989-1.031 | 1.006 | 0.015 |
| Simulated dataset IV | 9 | 0.982-1.033 | 1.009 | 0.019 |
| Simulated dataset V | 100 | 0.963-1.039 | 1.001 | 0.017 |

**Table S2. Descriptive statistics for λ_3_ values from SNP-by-genome interaction scans (model III) on all datasets.**

| **Dataset** | **Figure** | **Number of λ_3_ values** | **Range of λ_3_ values** | **Mean of λ_3_ values** | **Standard deviation of λ_3_ values** |
| --- | --- | --- | --- | --- | --- |
| FamHS | 1A | 81 | 0.860-1.336 | 1.017 | 0.071 |
| Simulated dataset I | 1B | 414 | 0.788-1.372 | 1.006 | 0.077 |
| Simulated dataset II | 1C | 414 | 0.790-1.514 | 1.011 | 0.077 |
| Simulated dataset III | 1D | 414 | 0.888-1.137 | 0.998 | 0.034 |
| Simulated dataset IV | 1E | 414 | 0.916-1.080 | 1.002 | 0.028 |
| Simulated dataset V | S7 | 1000 | 0.682-1.344 | 1.003 | 0.071 |


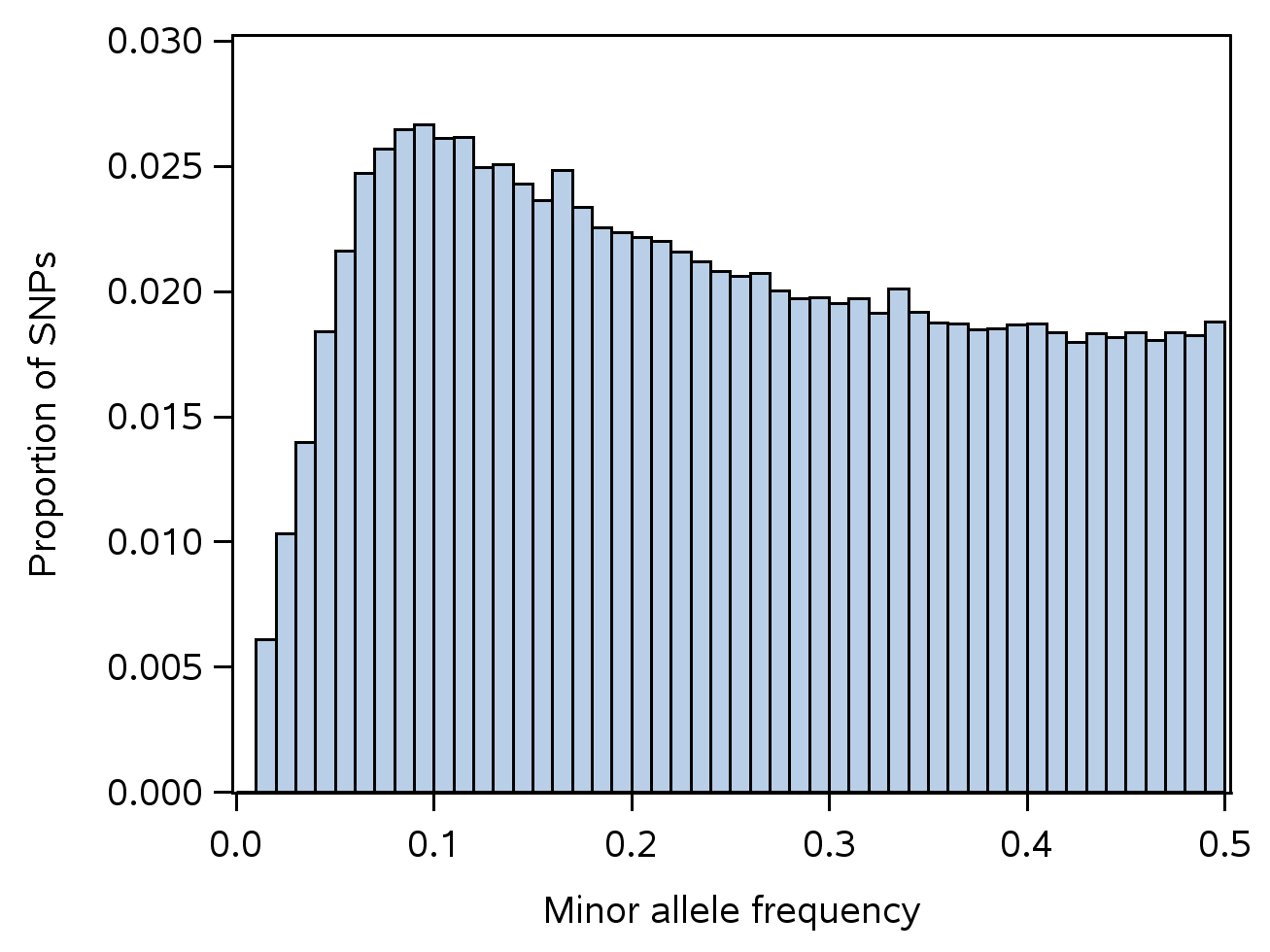


**Figure S1. MAF distribution of FamHS genotyped “non-repeating” SNPs (MAF≥1%) used in SNP-by-genome interaction scans (n=493,865 SNPs).**


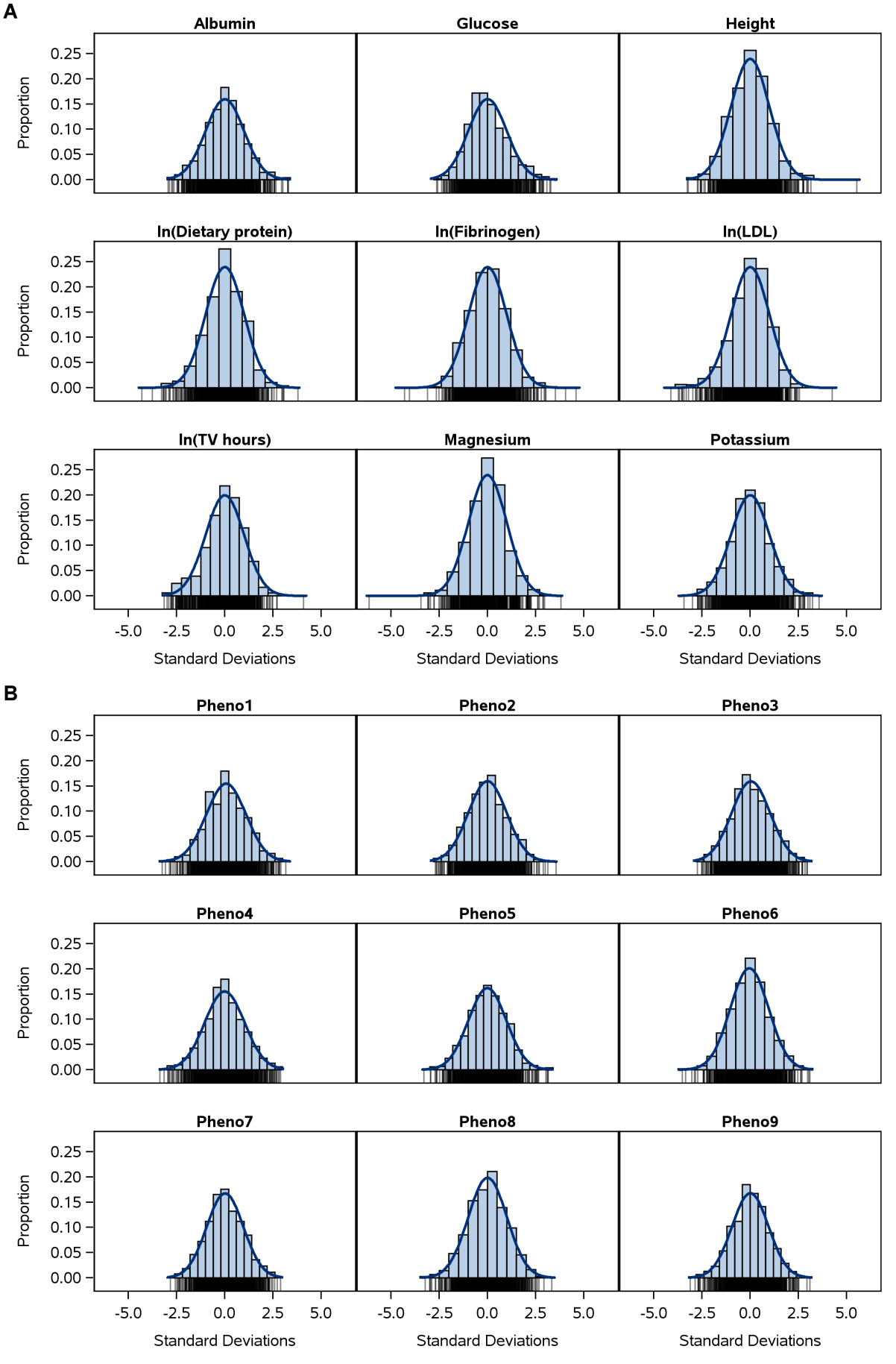


**Figure S2. Distributions of adjusted FamHS phenotypes (panel A) and simulated dataset II phenotypes (panel B).** Both datasets contained 1053 subjects. Simulated dataset II phenotypes were drawn from a standard normal distribution.


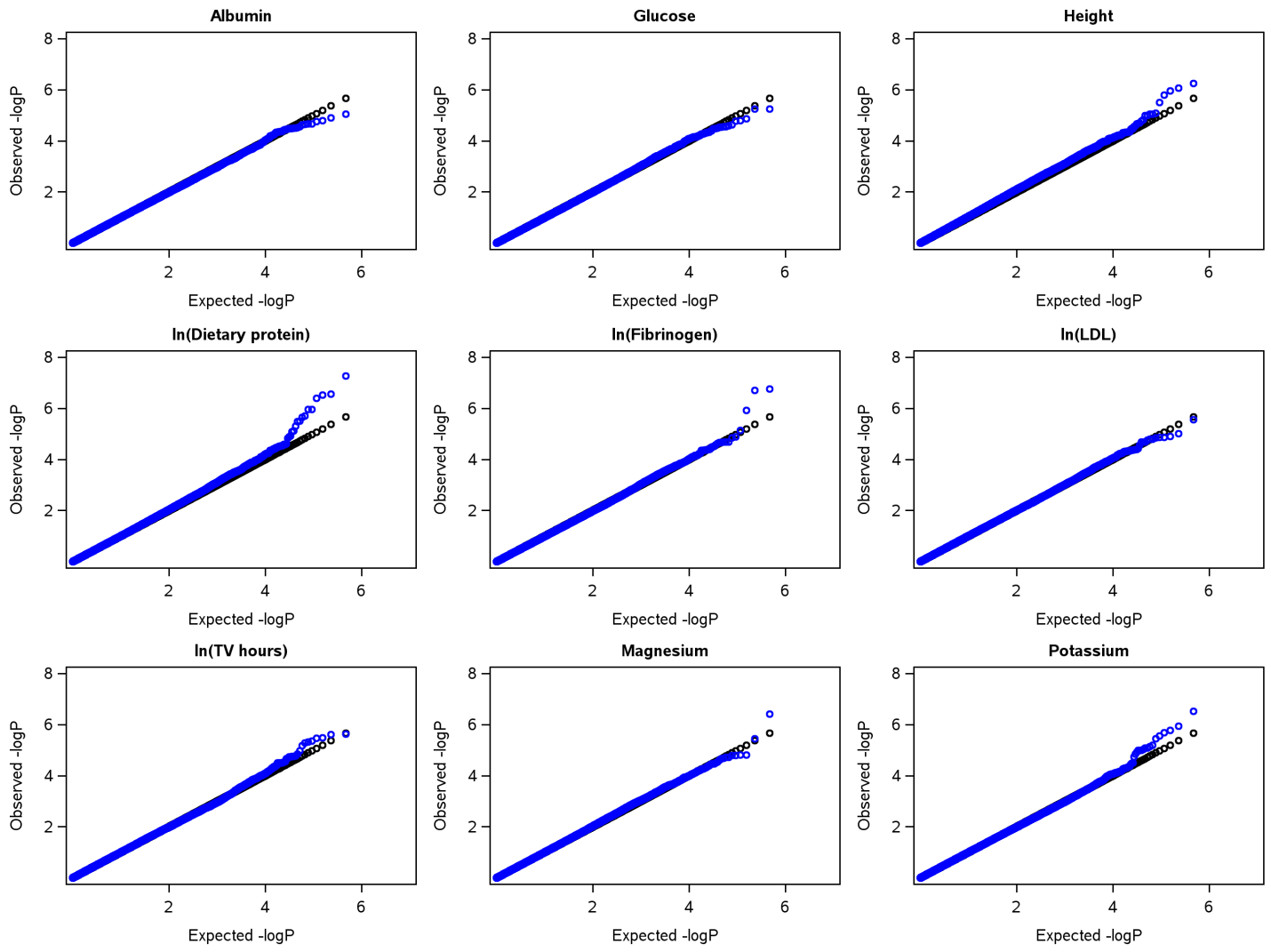


**Figure S3. Q-Q plots for main-effect-only scans (model I in Methods) performed on FamHS dataset for 9 phenotypes.** Blue indicates observed vs. expected -log_10_(p) values; black indicates the line y=x.


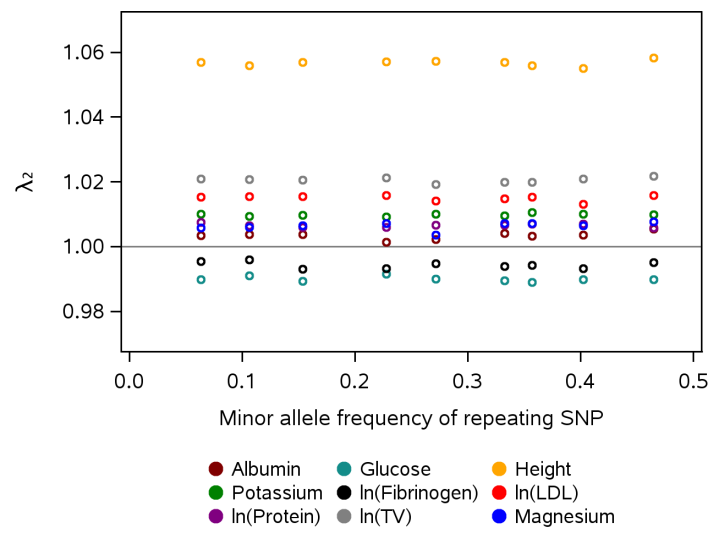


**Figure S4. Lambda corresponding to non-repeating SNP (λ_2_) in two-locus model without interaction term (model II in Methods) applied to 9 phenotypes in FamHS dataset.** Values are very similar to those obtained from main-effect-only scans on the same traits (Table 1).


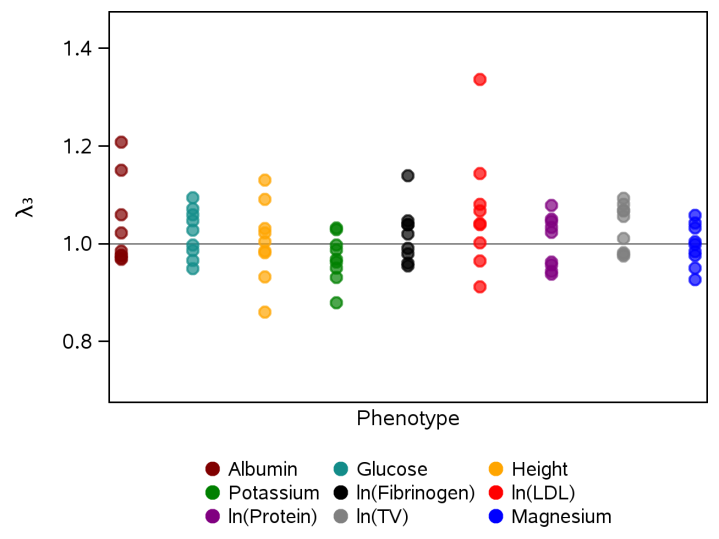


**Figure S5. Interaction-term lambda (λ_3_) from SNP-by-genome interaction scans in FamHS dataset plotted by phenotype.** Inflation and deflation of λ_3_ occurred for all 9 phenotypes.


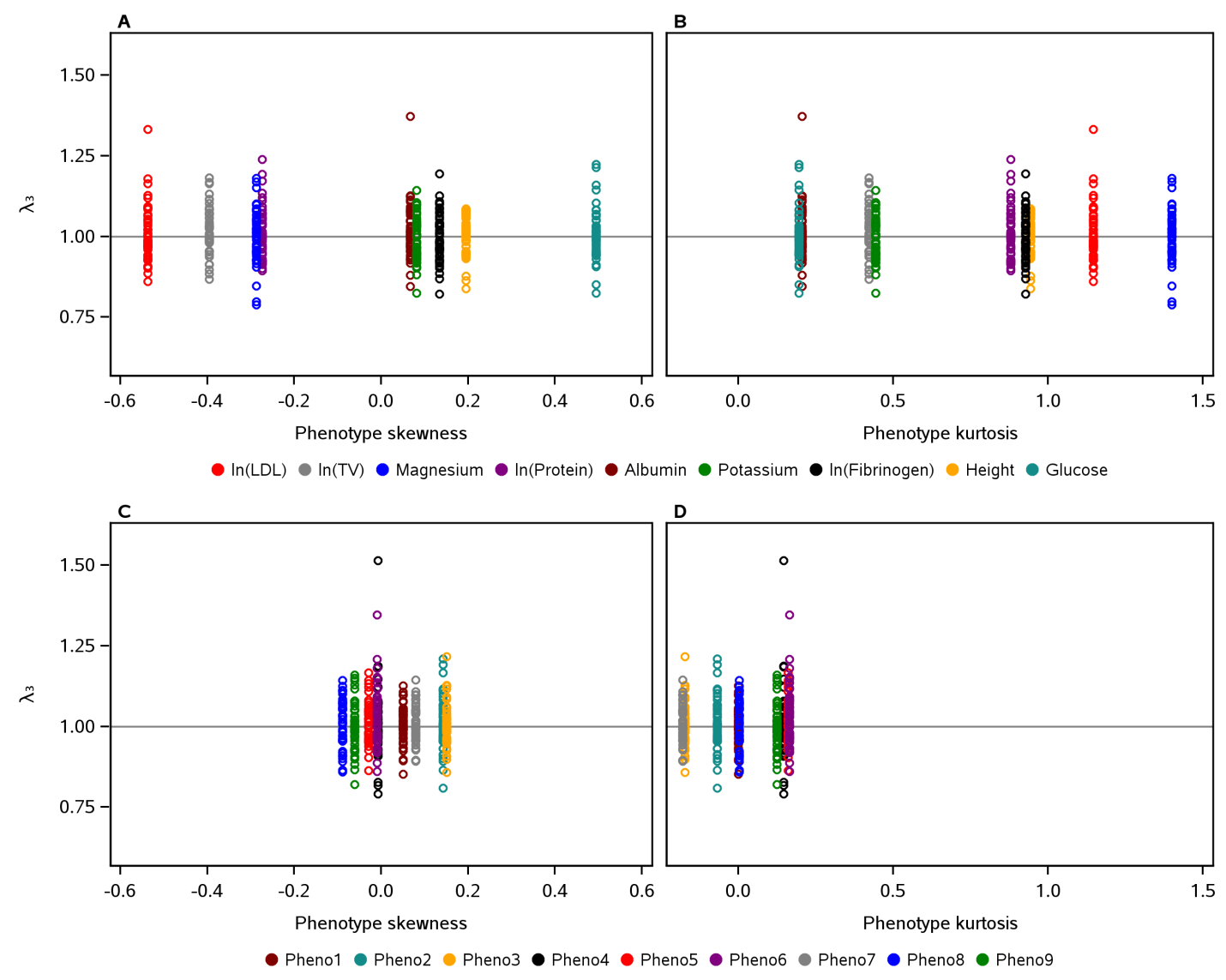


**Figure S6. Interaction-term lambda (λ_3_) from SNP-by-genome scans in simulated dataset I (panels A and B) and simulated dataset II (panels C and D) plotted by skewness and kurtosis of phenotype.** Simulated dataset I had FamHS phenotypes while simulated dataset II had phenotypes drawn from a standard normal distribution; both datasets had simulated genotypes and a sample size of 1053.


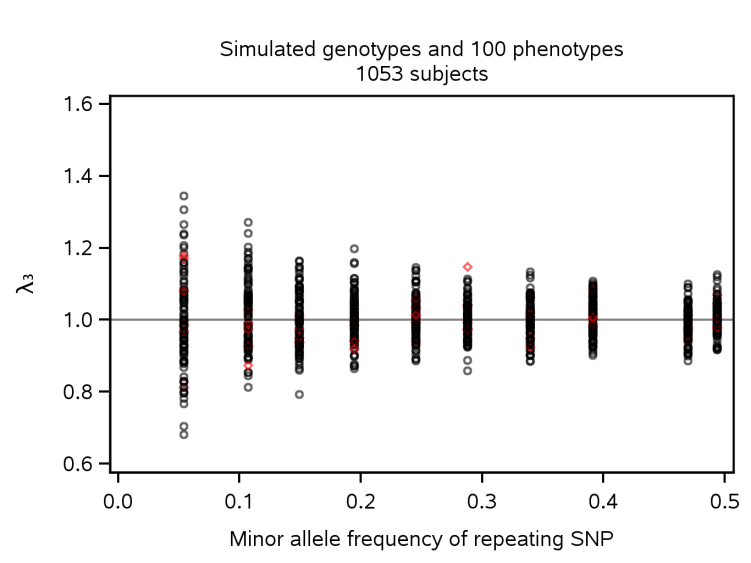


**Figure S7. Interaction-term lambda (λ_3_) from SNP-by-genome interaction scans on simulated dataset V plotted by MAF of repeating SNP.** Simulated dataset V had 100 phenotypes drawn from a standard normal distribution, simulated genotypes, and a sample size of 1053.


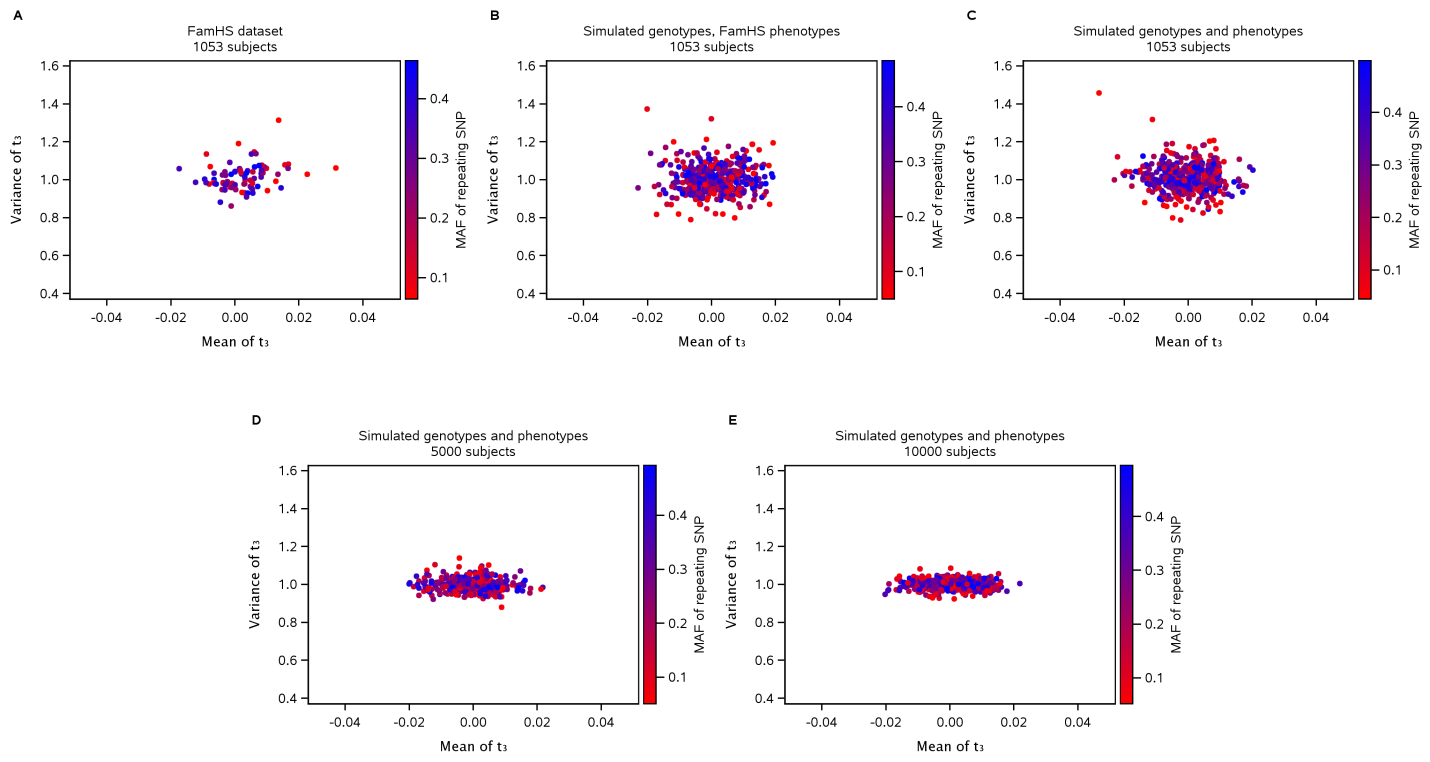


**Figure S8. Variances and means of interaction-term test statistic (t_3_) distributions from SNP-by-genome interaction scans.** The test statistic corresponding to the test of β_3_=0 in the product-term model (model III in Methods) is indicated by t_3_. Results for FamHS are shown in panel A; results for simulated datasets I-IV are shown in panels B-E, respectively.


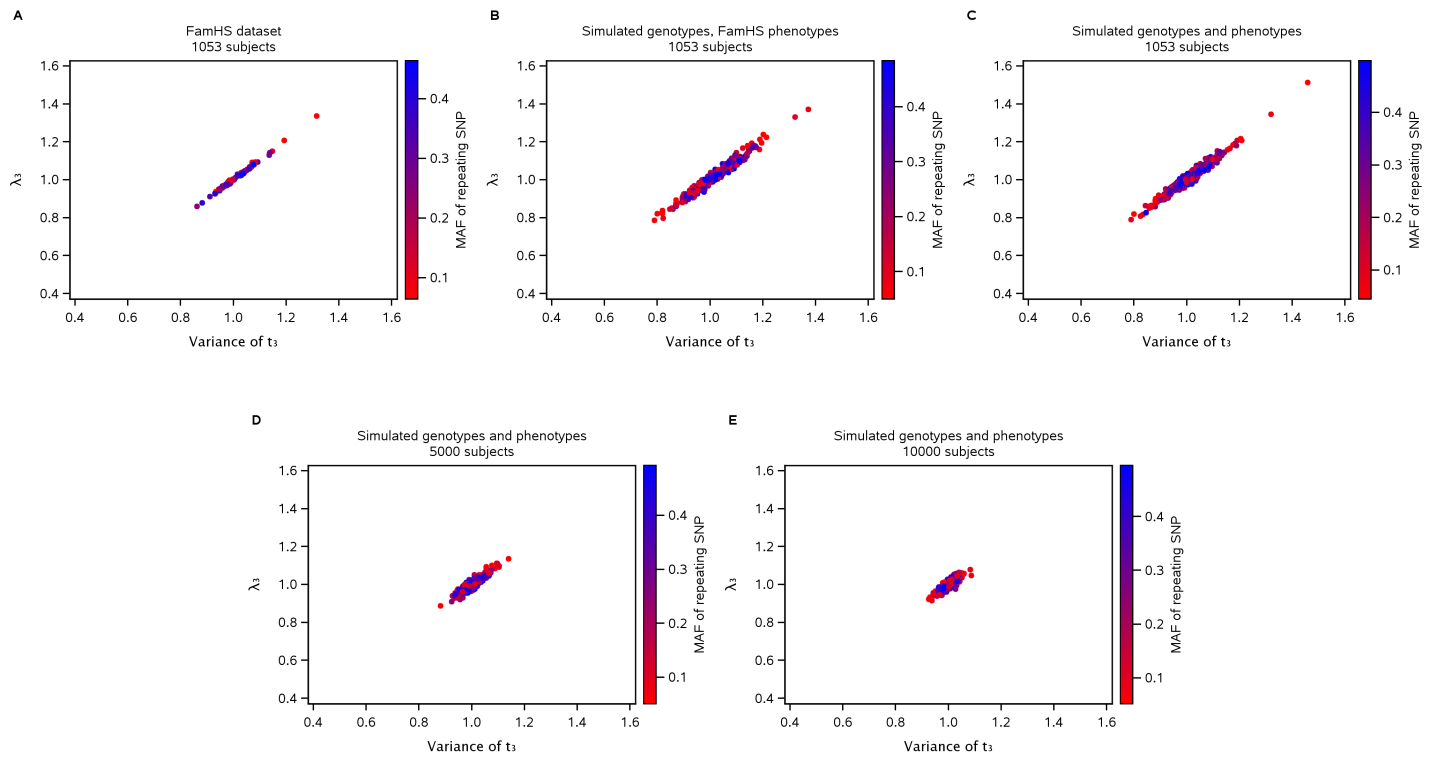


**Figure S9. Interaction-term lambda (λ_3_) plotted by variance of interaction-term test statistic distributions from SNP-by-genome interaction scans.** As in Fig. S8, the test statistic corresponding to the test of β_3_=0 in the product-term model is indicated by t_3_. Results for FamHS are shown in panel A; results for simulated datasets I-IV are shown in panels B-E, respectively. R^2^ values for a fitted regression line are (panel A) 0.99; (panel B) 0.97; (panel C) 0.97; (panel D) 0.84; and (panel E) 0.77.


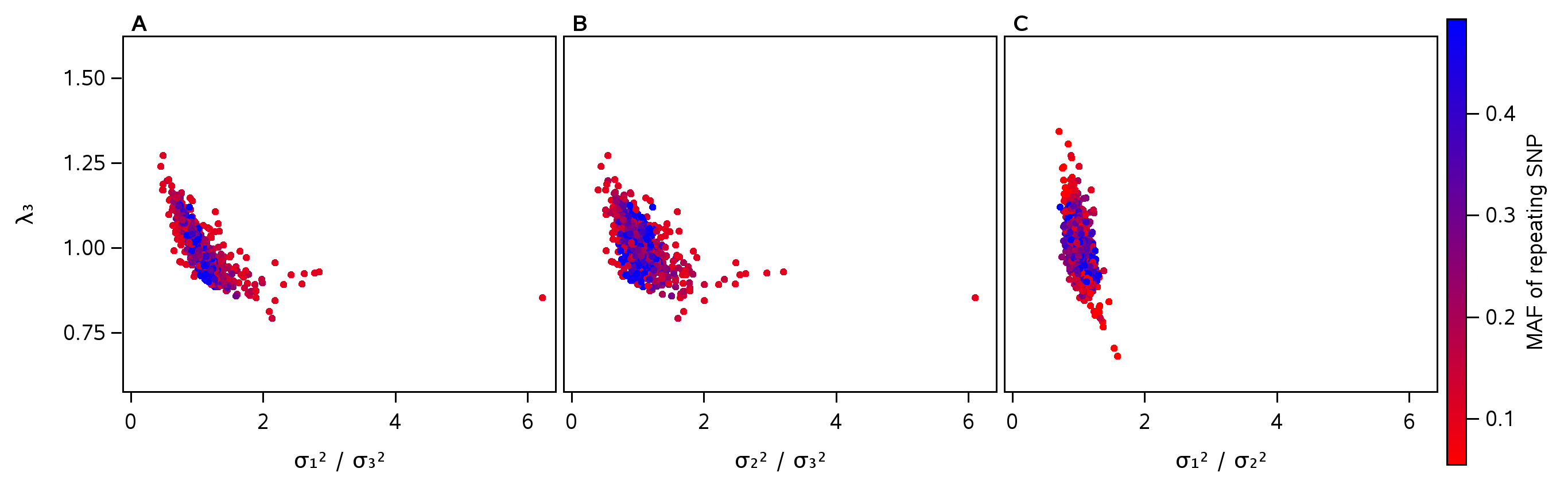


**Figure S10. Interaction-term lambda (λ_3_) from SNP-by-genome interaction scans on simulated dataset V plotted by repeating-SNP genotypic variance ratios.** As in Fig. 2, plotted on the x-axes are the genotypic variance ratios of the largest to smallest genotype class (panel A), the middle to smallest genotype class (panel B), and the largest to the middle genotype class (panel C) of the repeating SNPs used in the SNP-by-genome interaction scans. Interaction-term lambda values from all 1000 SNP-by-genome scans performed on this dataset (10 repeating SNPs, 100 phenotypes) are indicated by the y-axis.


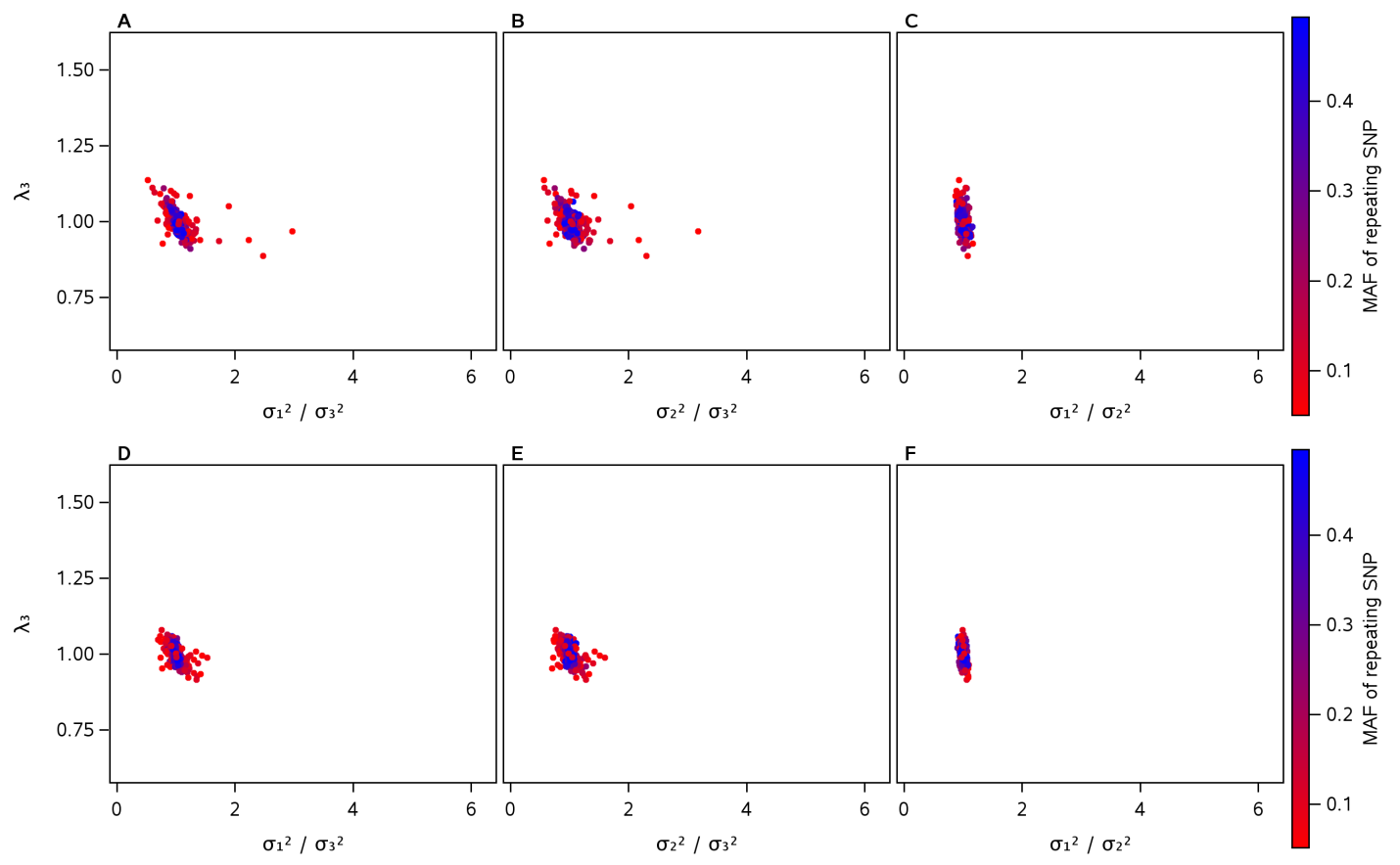


**Figure S11. Interaction-term lambda (λ_3_) from SNP-by-genome interaction scans on simulated datasets III and IV plotted by repeating-SNP genotypic variance ratios.** Simulated dataset III (panels A-C) had 5000 subjects and simulated dataset IV (panels D-F) had 10,000 subjects. As in Fig. 2, plotted on the x-axes are the genotypic variance ratios of the largest to smallest genotype class (panels A and D), the middle to smallest genotype class (panels B and E), and the largest to the middle genotype class (panels C and F) of the repeating SNP used in the SNP-by-genome interaction scan. Interaction-term lambda values from all 414 SNP-by-genome scans performed on each of these datasets (46 repeating SNPs, 9 phenotypes) are indicated by the y-axis.


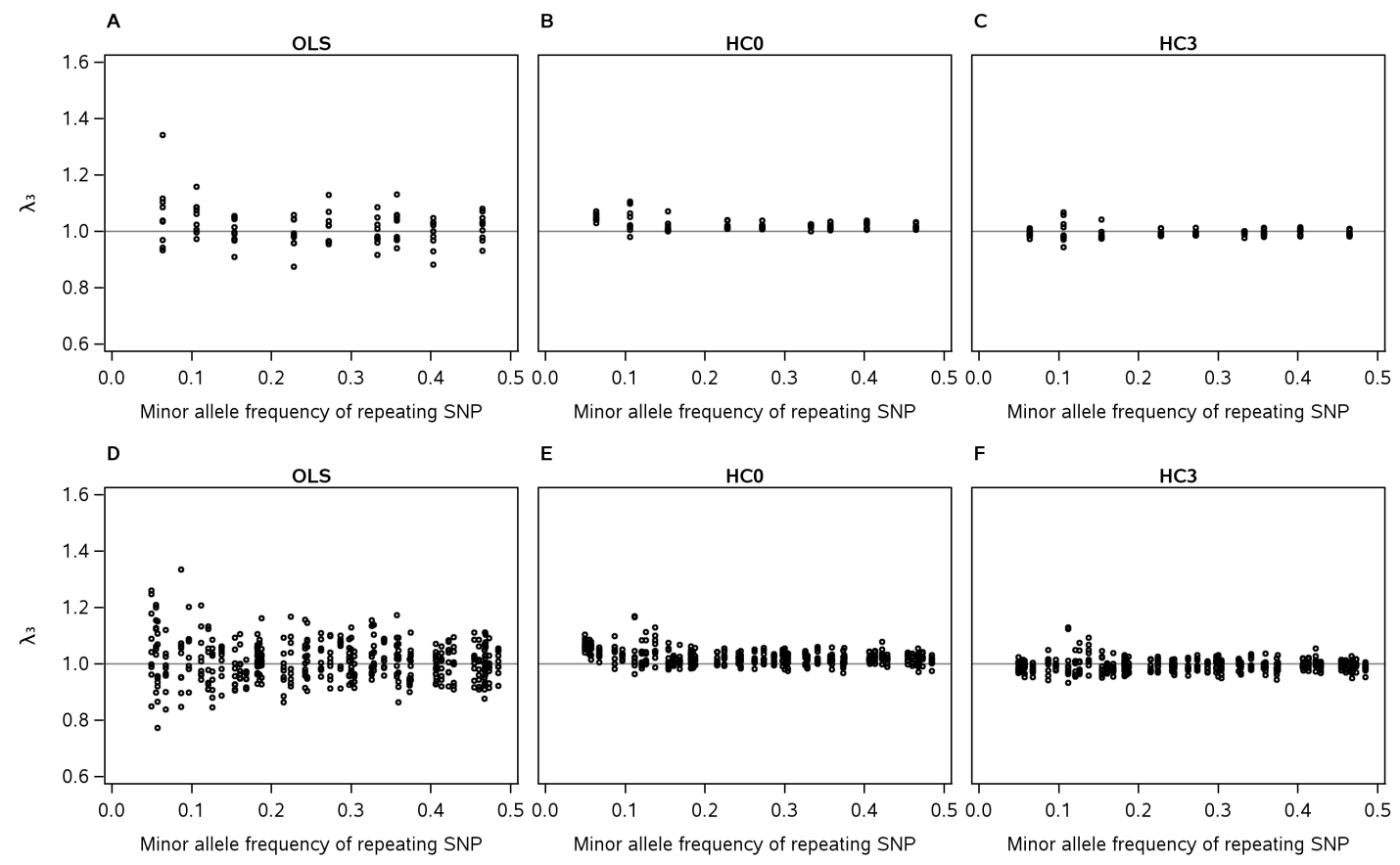


**Figure S12. Comparison of interaction-term lambda (λ_3_) distributions obtained using three types of standard errors in SNP-by-genome interaction scans on FamHS dataset and simulated dataset I.** Ordinary least squares standard errors were used in panels A and D; HC0 standard errors were used in panels B and E; and HC3 standard errors were used in panels C and F. Analyses for FamHS are shown in panels A-C; analyses for simulated dataset I are shown in panels D-F. Subjects in two-locus genotype classes with fewer than 5 subjects were dropped before analyses were performed, which distinguishes panels A and D from Fig. 1 panels A and B, respectively.


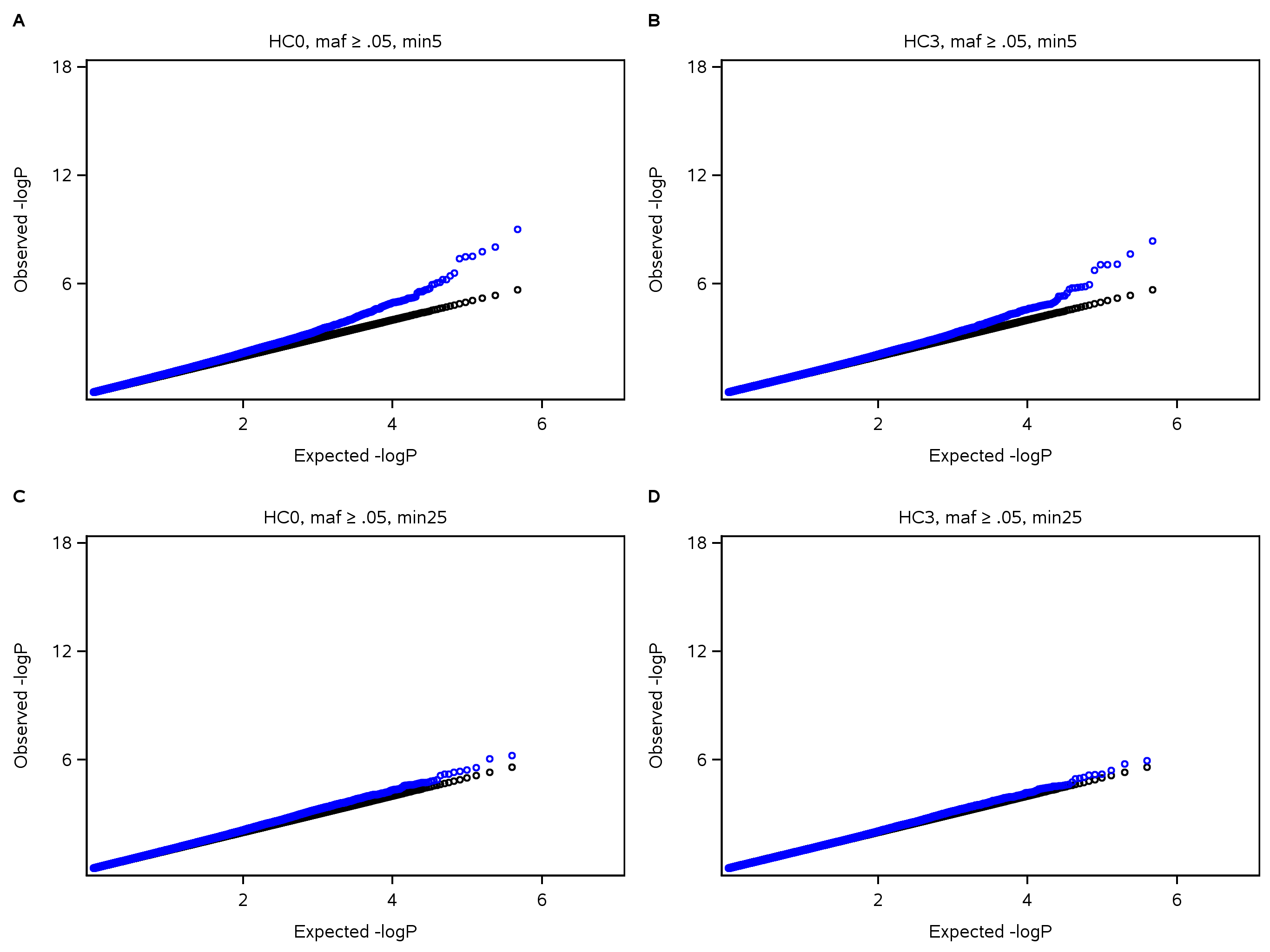


**Figure S13. Q-Q plots from SNP-by-genome interaction scans using HC0 and HC3 standard errors and two different cell-count thresholds.** As in Fig. 4, SNP-by-genome interaction scan was in FamHS dataset for repeating SNP 1 and the phenotype of ln(LDL cholesterol). HC0 standard errors were used in panels A and C, and HC3 in panels B and D. A MAF threshold of 0.05 was used for the non-repeating SNPs, with an additional requirement that all populated two-locus genotype classes have at least 5 subjects (panels A and B) or 25 subjects (panels C and D). Subjects below the minimum cell count were excluded before analysis. Blue indicates observed vs. expected -log_10_(p) values; black indicates the line y=x.


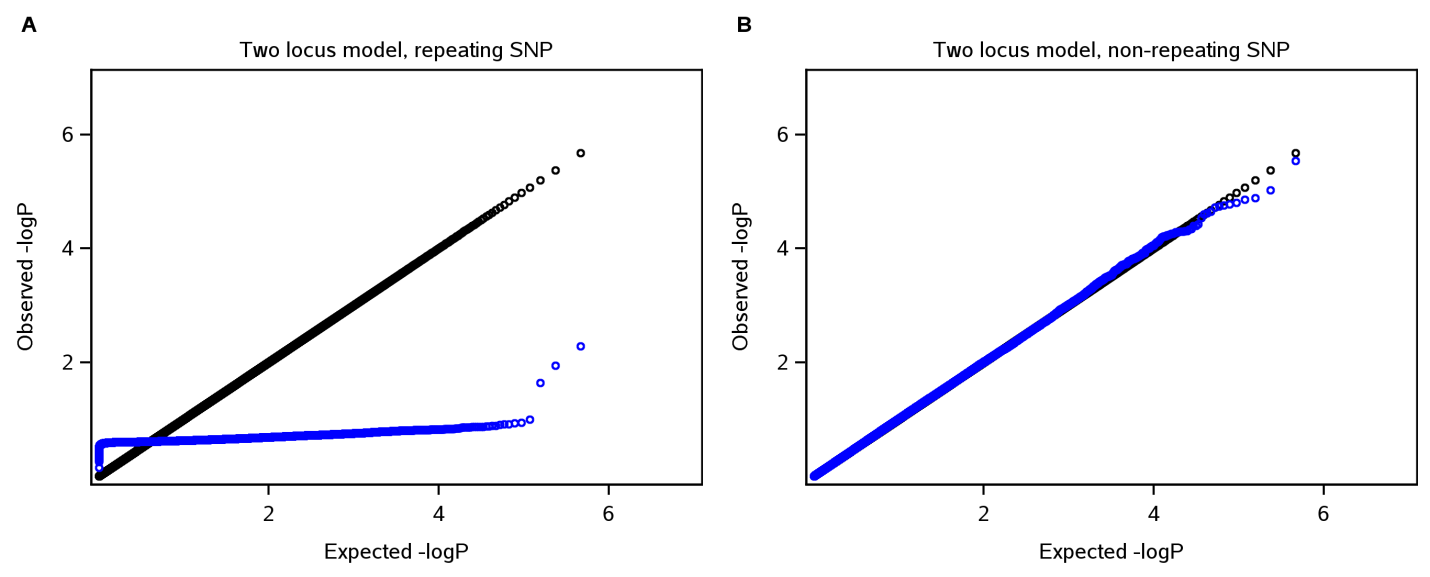


**Figure S14. Examples of Q-Q plots from two-locus model without interaction term (model II in Methods), applied to the phenotype of ln(LDL Cholesterol) in FamHS dataset.** Q-Q plots correspond to the repeating SNP (panel A) and the non-repeating SNP (panel B). When a product term is added to a two-locus model, its Q-Q plot can be expected to show properties of both conceptual extremes even in the absence of bias because the product term is correlated from test to test within a scan due to collinearity of the product term with its component terms (one of which repeats from test to test). Blue indicates observed vs. expected -log_10_(p) values; black indicates the line y=x.


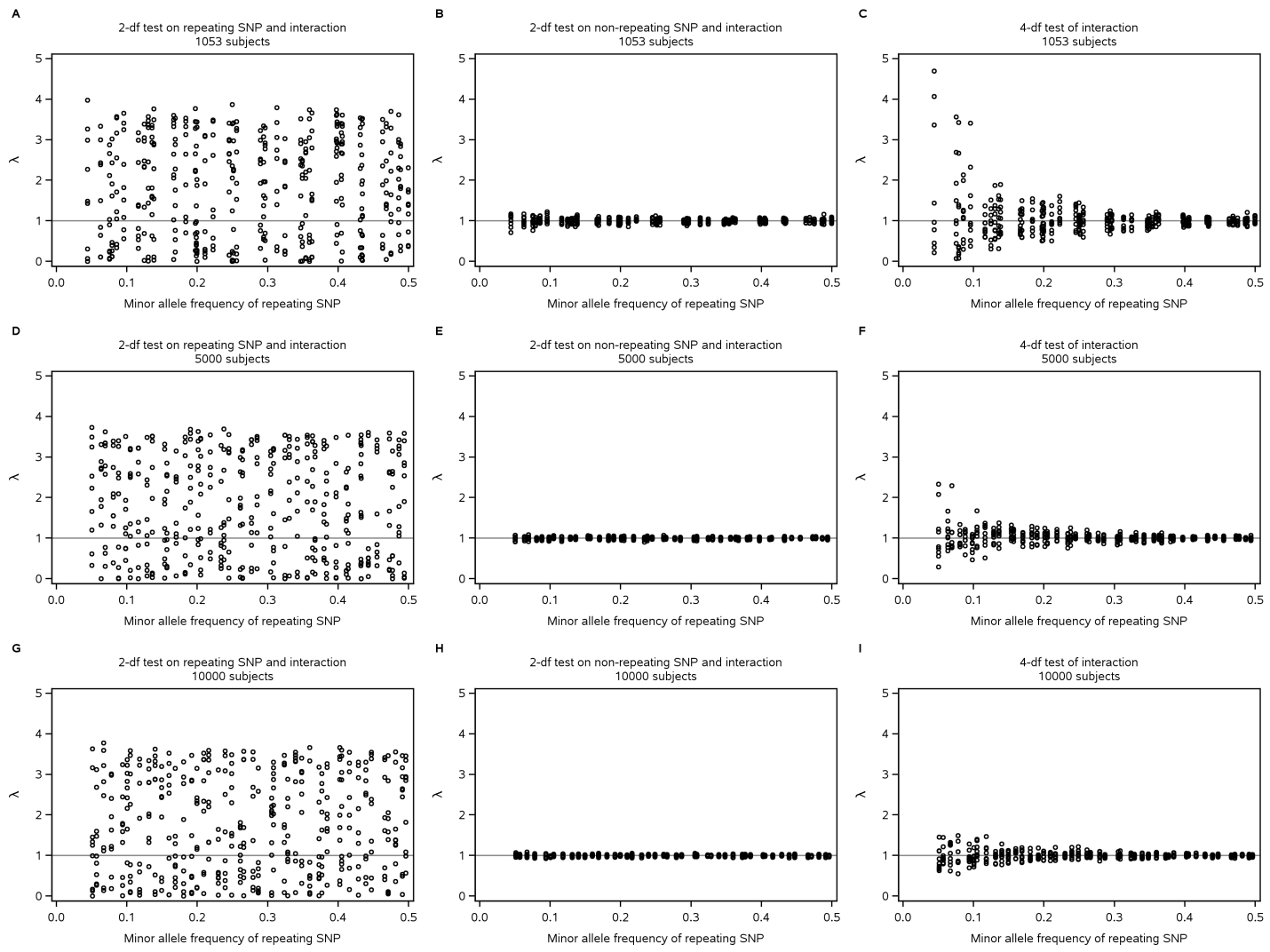


**Figure S15. Lambda values from 2- and 4-df SNP-by-genome interaction scans on simulated datasets II-IV plotted by MAF of repeating SNP.** Results for simulated dataset II (1053 subjects) are shown in panels A-C, results for simulated dataset III (5000 subjects) are shown in panels D-F, and results for simulated dataset IV (10,000 subjects) are shown in panels G-I. In panels A, D, and G, the 2-df test was on the repeating SNP and the product term in model III (see Methods). In panels B, E, and H, the 2-df test was on the non-repeating SNP and the product term. In panels C, F, and I, the 4-df test was on the four product terms in model IV (see Methods). For all panels, the λ value for each scan was calculated by finding the Χ^2^_1_ values corresponding to the *F*-test p-values, and dividing the median Χ^2^_1_ value by the median of the Χ^2^_1_ distribution (0.455). For the 4-df test, collinearities that arose among terms in the model at low MAF of the repeating SNP and low sample size led some of the 20,000 tests to drop out; at a sample size of 1053 subjects, the constant SNP with the second-lowest MAF did not produce any results, while the remainder of the 46 constant SNPs gave a range of 1621-18666 results. At a sample size of 5000, this range was 11124-19975, and at a sample size of 10000 it was 13726-20000. For all 6 sets of 2-df tests, no tests dropped out, so each λ value summarizes all 20,000 tests.


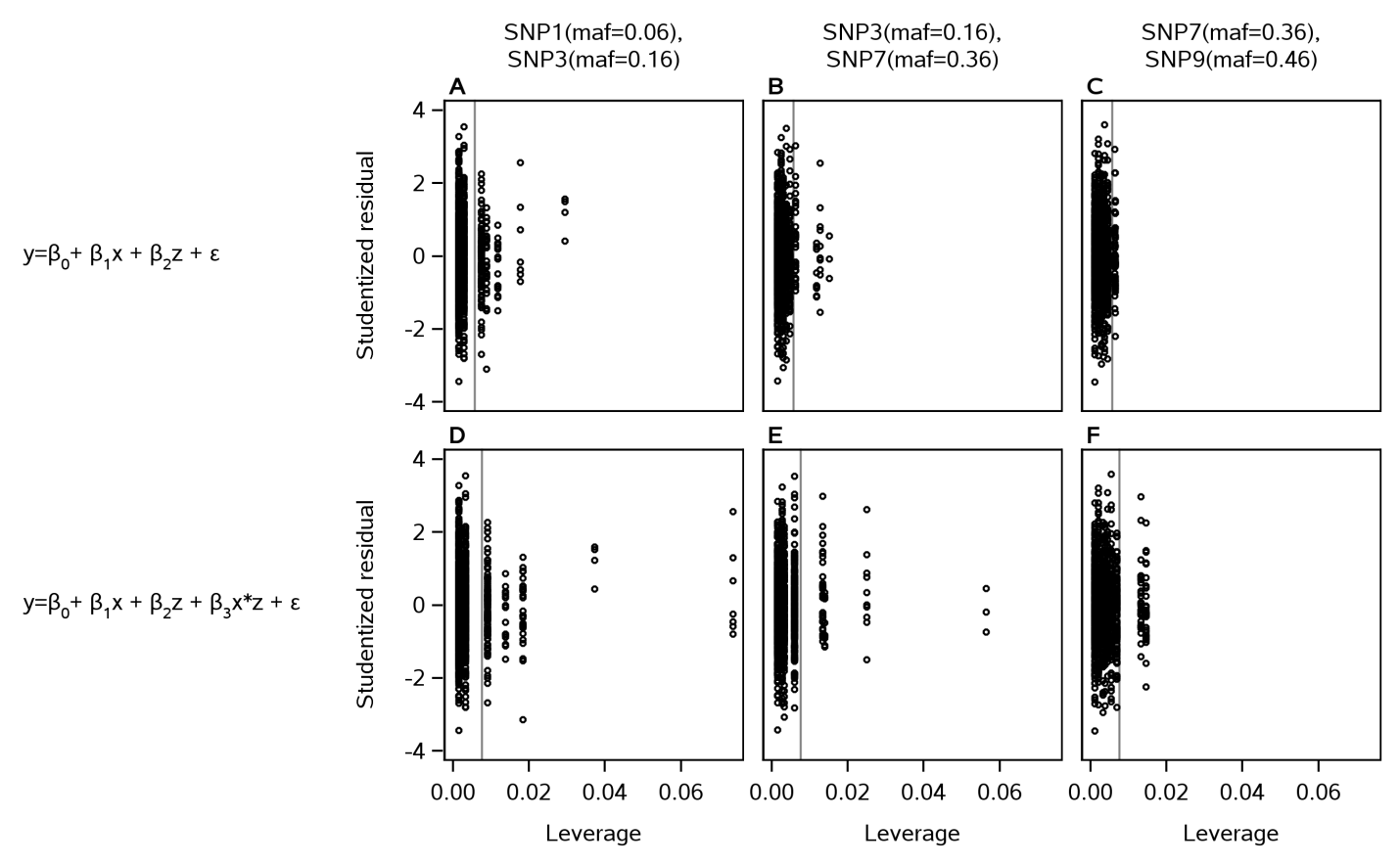


**Figure S16. Examples of residual-by-leverage plots for a two-locus model and a product-term model, applied to the phenotype of serum potassium in FamHS dataset.** Pairs of SNPs from the list of 9 repeating SNPs were used because they were already characterized (Table 2). Reference lines indicate 2p/n, the cutoff suggested by Belsley, Kuh and Welsch [1980] to designate points of high leverage where p is the number of parameters and n is the sample size. Residual-by-leverage plots illustrate that while both models separate the subjects into the same two-locus genotype classes, subjects are drawn to points of higher leverage by the product-term model (Panels D-F) than by the two-locus model (Panels A-C).
